# Dynamic association of the intramembrane proteases SPPL2a/b and their substrates with tetraspanin-enriched microdomains

**DOI:** 10.1016/j.isci.2023.107819

**Published:** 2023-09-04

**Authors:** Nadja Leinung, Torben Mentrup, Mehul Patel, Tom Gallagher, Bernd Schröder

**Affiliations:** 1 Institute of Physiological Chemistry, Technische Universität Dresden, Dresden, Germany; 2 Department of Microbiology and Immunology, Loyola University Chicago, Maywood, IL, USA

**Keywords:** Biological sciences, Molecular biology, Molecular interaction

## Abstract

Signal peptide peptidase-like 2a and b (SPPL2a/b) are aspartyl intramembrane proteases and cleave tail-anchored proteins as well as N-terminal fragments (NTFs) derived from type II-oriented transmembrane proteins. How these proteases recruit substrates and cleavage is regulated, is still incompletely understood. We found that SPPL2a/b localize to detergent-resistant membrane (DRM) domains with the characteristics of tetraspanin-enriched microdomains (TEMs). Based on this, association with several tetraspanins was evaluated. We demonstrate that not only SPPL2a/b but also their substrates tumor necrosis factor (TNF) and CD74 associate with tetraspanins like CD9, CD81, and CD82 and/or TEMs and analyze the stability of these complexes in different detergents. CD9 and CD81 deficiency has protease- and substrate-selective effects on SPPL2a/b function. Our findings suggest that reciprocal interactions with tetraspanins may assist protease-substrate encounters of SPPL2a/b within the membrane. Beyond SPP/SPPL proteases, this supports previous concepts that tetraspanins facilitate membrane-embedded proteolytic processes.

## Highlights

• Signal peptide peptidase-like 2a/b associate with tetraspanin-enriched domains
• TEM association of SPPL2b is promoted by complex formation with CD9
• Recruitment of SPPL2a into tetraspanin complexes is supported by its substrate TNF
• Cleavage of CD74 N-terminal fragments by SPPL2a is impaired in the absence of CD81

## Graphical abstract

**Figure:**
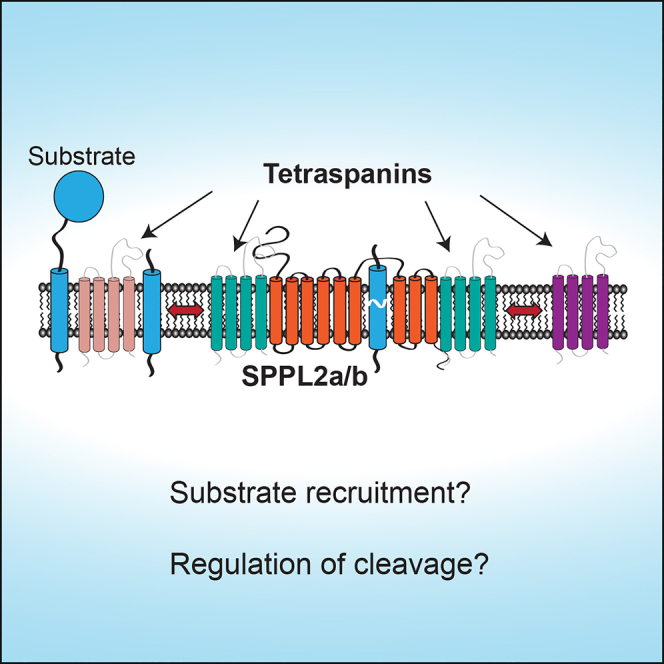

## Introduction

Intramembrane proteases (I-CLIPs) cleave substrate proteins within the hydrophobic environment of the phospholipid bilayer.^1^^,^^2^ In most cases, substrate proteins are single-span transmembrane proteins—depending on the protease either in type I or II orientation.^1^^,^^2^ Some intramembrane proteases require a pre-processing of the substrate’s ectodomains by membrane-bound sheddases, e.g., of the disintegrin and metalloproteinase (ADAM) family, or endosomal proteases before cleavage within the transmembrane segment can take place.^3^ Such sequential processing is referred to as regulated intramembrane proteolysis (RIP) and requires that substrates are transferred between the involved enzymes.

I-CLIPs can be classified according to their catalytic mechanism into metallo-, serine, glutamyl, and aspartyl proteases.^1^^,^^2^ The latter are constituted by the presenilins, the catalytic subunits of the γ-secretase complex,^4^ as well as signal peptide peptidase (SPP) and its four homologous SPP-like (SPPL) proteases.^5^^,^^6^ The SPPL proteases SPPL2a and SPPL2b are predominantly localized in the endolysosomal system and at the plasma membrane, respectively. Both cleave N-terminal fragments (NTFs) derived from type II transmembrane proteins in an RIP sequence^7^ as well as tail-anchored proteins,^8^ which are direct substrates. *In vivo*-validated substrates of SPPL2a and/or SPPL2b, which are associated with specific phenotypes of SPPL2a/b-deficient cells and/or mice, are the invariant chain of the MHCII complex (CD74),^9^^,^^10^ the lectin-like oxidized LDL receptor 1 (LOX-1)^11^ and the fungal pattern recognition receptor Dectin-1.^12^ Accumulation of NTFs from these substrates directly or indirectly alters certain trafficking or signaling pathways. The absence of SPPL2a and/or SPPL2b impairs the development and function of B lymphocytes^9^^,^^13^^,^^14^ as well as dendritic cells^15^^,^^16^ and promotes the development of atherosclerotic plaques.^11^ In addition, cleavage of the SNARE (soluble N-ethylmaleimide-sensitive-factor attachment receptor) proteins VAMP1-4 has been confirmed *in vivo* based on an accumulation of these proteins in several tissues of SPPL2a/b-deficient mice.^8^ However, the functional implications of this are currently unknown. Altogether, substrate selection by these proteases as well as potential regulatory mechanisms of SPPL2a/b are currently not well understood.^7^

In general, as compared to proteolysis in an aqueous environment, intramembrane proteases face the challenge that diffusion of both the enzymes and the substrates are restricted to the plane of the membrane. Since its introduction in 1972, the fluid mosaic model of membranes^17^ has been challenged by concepts that proteins and lipids are not as homogenously distributed as suggested by this model. Interactions of specific lipids like cholesterol and sphingolipids have been proposed to be the organizing principle of membrane microdomains called “lipid rafts”.^18^ Despite the demonstration of lipid phase separations in model membranes,^19^ a visualization of lipid rafts *in situ* has remained challenging for a long time.^20^ However, advances in microscopy and spectroscopy have allowed a more direct proof of membrane compartmentalization.^21^^,^^22^ Specific sets of membrane proteins are assumed to dynamically associate with lipid rafts.^18^

In addition to lipid partitioning, specific proteins can contribute to the lateral organization of cellular membranes by inducing formation of nano- or microscale microdomains.^23^ One protein family with these properties is tetraspanins, which comprise more than 30 members in humans.^23^ Tetraspanins exhibit a characteristic topology with four transmembrane domains and one large extracellular/luminal loop (LEL) with a conserved cysteine motif^24^ and have been implicated in various processes including signaling,^24^^,^^25^ trafficking,^26^ and immune cell function.^27^^,^^28^ They are able to establish lateral interactions with other tetraspanin molecules.^23^ In particular, the LEL and the first two transmembrane segments seem to be involved in this.^23^ By this means, tetraspanins form tetraspanin-enriched microdomains (TEMs) also referred to as the tetraspanin web.^29^^,^^30^^,^^31^ Using super-resolution microscopy, the diameter of tetraspanin clusters has been estimated to be ∼100 nm.^32^ Importantly, tetraspanins can also interact with partner proteins like transmembrane receptors, adhesion molecules, enzymes, signaling proteins etc., which thereby become associated with TEMs. This is the basis for the aforementioned wide-spread functional impact of tetraspanins.

Despite being most intensively studied in the plasma membrane, formation of subdomains within membranes is not restricted to this compartment.^33^ Lipid rafts have also been reported in the endolysosomal system.^34^^,^^35^ Furthermore, several tetraspanins have documented roles there.^26^ Since the γ-secretase complex, which is mechanistically related to SPP/SPPL proteases, has been reported to be associated with different types of membrane subdomains^36^^,^^37^^,^^38^ and several membrane-embedded proteases e.g., ADAM10 interact with tetraspanins,^39^ we aimed to analyze these aspects for SPPL2a/b. We show that these proteases associate with TEMs and are present in complexes containing the tetraspanins CD9, CD81 and CD82. Loss of CD9 or CD81 differentially impacts microdomain association and/or substrate cleavage of SPPL2a/b. We also demonstrate association of the SPPL2a/b substrate TNF with tetraspanins, which can support recruitment of the proteases to these complexes. Our results suggest that tetraspanin associations of both the intramembrane protease SPPL2a/b as well as their substrates assist in facilitating encounters of both partners and by this means substrate cleavage.

## Results

### SPPL2a/b are localized in DRMs with the characteristics of TEMs

**Figure 1:**
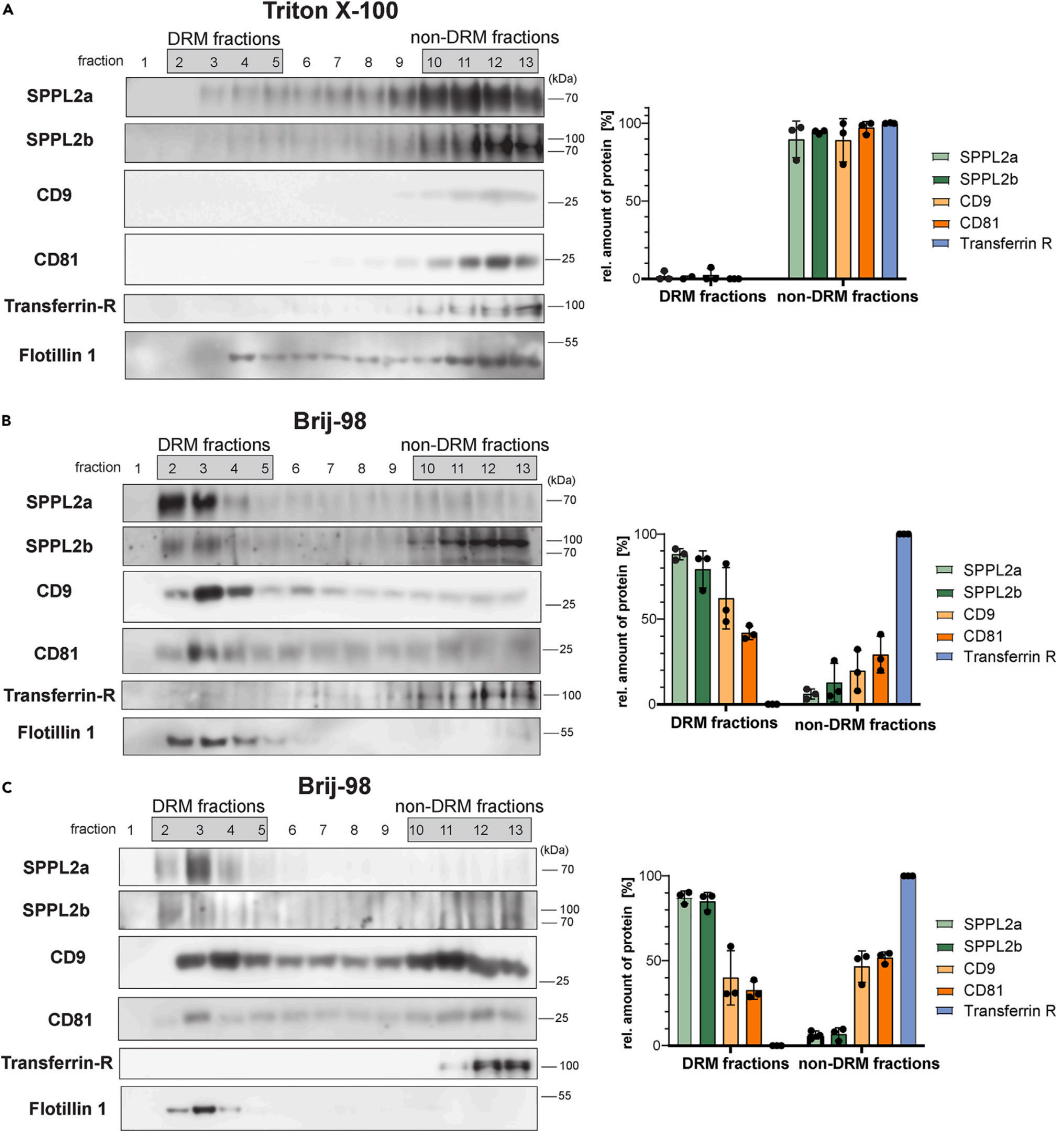
SPPL2a/b are associated with detergent-resistant membranes (DRM) (A and B) Mouse embryonic fibroblasts (MEF) from wild type mice were solubilized with 1% Triton X-100 (A) or 1% Brij-98 (B). Lysates were separated using discontinuous sucrose density gradients at 263,627 x g_max_ for 16 h at 4°C. Thirteen fractions of 1 mL were collected starting from the top. Equal volumes of each fraction were analyzed by Western blotting. DRMs were recovered in fractions 2–5, whereas non-DRM-associated proteins remained in the sample loading area in fractions 10–13. (C) The DRM-association of SPPL2a/b was further examined in bone marrow-derived dendritic cells (BMDC) from C57BL/6 wild-type mice which were solubilized with 1% Brij-98, subjected to density centrifugation and analyzed by Western blotting as described previously. The transferrin receptor (Transferrin-R, anti-Transferrin Receptor, Abcam clone ab84036) and flotillin-1 were detected as controls. Band intensities of SPPL2a/b, CD9, CD81, and transferrin receptor were densitometrically quantified in all fractions. The intensities observed in DRM fractions (fraction 2–5) and non-DRM fractions (fraction 10–13) were combined, respectively. These combined intensities are depicted as proportion (%) of the total amount of the respective protein detected across the entire gradient. Mean ± S.D., N = 3, n = 3.

Isolation of detergent-resistant membranes (DRMs) by density gradient centrifugation following extraction with non-ionic detergents has been widely used as a screening approach to assess the association of proteins with membrane subdomains like lipid rafts or TEMs.^19^^,^^29^ Therefore, we extracted wild-type MEFs that express significant amounts of both SPPL2a and SPPL2b either with 1% Triton X-100 (Figure 1A) or 1% Brij-98 (Figure 1B). Lysates were layered beneath a discontinuous sucrose step gradient, from which fractions were recovered from top to bottom after overnight ultracentrifugation for Western blot analysis. Following Triton X-100 extraction, SPPL2a and SPPL2b were predominantly detected in the bottom fractions 10–13 corresponding to the sample loading zone of the gradient (Figure 1A). However, when Brij-98 was used (Figure 1B) SPPL2a was present exclusively in the top fractions containing floating DRMs. In addition, a major fraction of SPPL2b was redistributed to these fractions in this detergent. As a control, we visualized the distribution of the established lipid raft marker flotillin-1^40^^,^^41^ and the transferrin receptor,^41^^,^^42^^,^^43^ which is reported to be absent from lipid rafts or other types of DRMs. We wanted to scrutinize these observations in an independent cell type and repeated this analysis with BMDCs from wild-type mice (Figure 1C). Again, SPPL2a and SPPL2b were completely or to a major extent, respectively, recovered in the DRM fractions after lysis with Brij-98, where flotillin-1 was present but transferrin receptor was absent. No flotation of SPPL2a/b was observed when cells were extracted with Triton X-100 (data not shown). Thus, the findings from the MEF cells were recapitulated in BMDCs supporting an association of SPPL2a and SPPL2b with certain membrane subdomains, which are resistant to Brij-98, but disrupted by Triton X-100. Different types of membrane microdomains, including lipid rafts and TEMs, can exhibit detergent resistance.^29^^,^^44^ Therefore, a more specific characterization of the involved protein and/or lipid interactions of a DRM-associated protein is required to define the complexes involved. In general, the DRM composition differs depending on the used detergent.^44^ We also tested solubilization with CHAPSO (Figure S1), which had also been used for the analysis of γ-secretase.^38^ Following CHAPSO extraction, SPPL2a was still detected in the top fractions of the sucrose gradient, however, to a lesser degree than following Brij-98 extraction. We failed to observe flotation of SPPL2b under these conditions.

Lipid-organized domains are considered to be stable in the presence of Triton X-100.^45^^,^^46^ By contrast, interactions between tetraspanins within TEMs were reported to be more labile being disrupted by Triton X-100 but preserved in the presence of different Brij variants^29^ and partially in CHAPSO as seen for γ-secretase.^38^ Along this line, major fractions of the CD9 and CD81 pools were recovered in DRM fractions following extraction with Brij-98 (Figures 1B and 1C). Flotation of these tetraspanins was less prominent in CHAPSO (Figure S1) and completely abolished in Triton X-100 (Figure 1A), which was very similar to the pattern observed for SPPL2a/b. Therefore, the observed properties of SPPL2a/b could indicate an association with TEMs.

### SPPL2a/b co-purify with the tetraspanins CD9, CD81, and CD82

**Figure 2:**
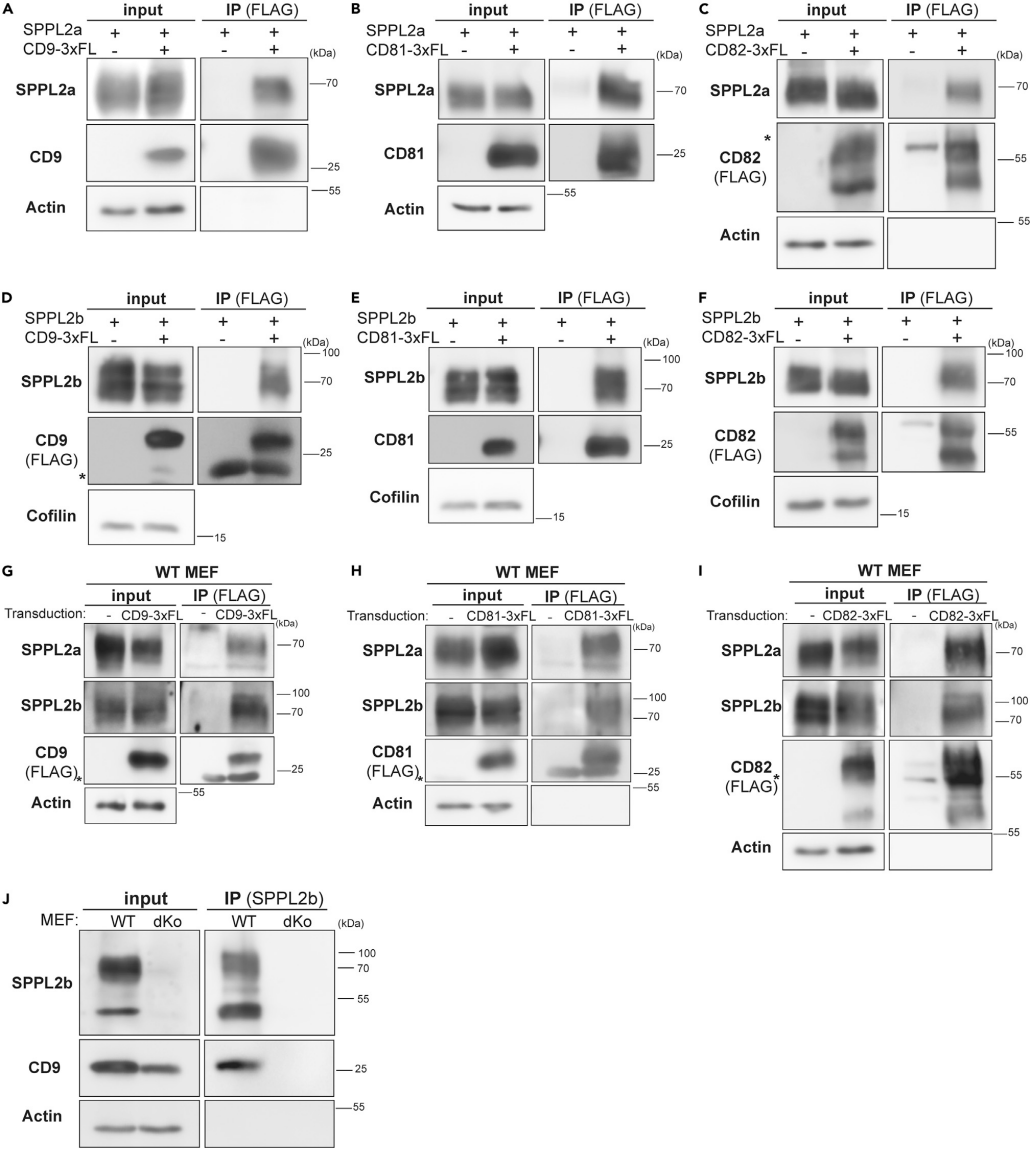
SPPL2a/b co-purify with the tetraspanins CD9, CD81, and CD82 (A–F) Transient overexpression of either SPPL2a-Myc with or without CD9-3xFLAG (A), CD81-3xFLAG (B), and CD82-3xFLAG (C) or SPPL2b-Myc with or without CD9-3xFLAG (D), CD81-3xFLAG (E), and CD82-3xFLAG (F) in HEK293 cells. (G–I) Additionally, wild-type MEF cells stably overexpressing CD9-3xFLAG (G), CD81-3xFLAG (H), or CD82-3xFLAG (I) were used for immunoprecipitation experiments. HEK and MEF cells were lysed with 1% Brij-98 and triple FLAG-tagged (3x-FL) CD9, CD81, or CD82 were immunoprecipitated using FLAG antibody-conjugated beads. MEF lysates were subjected to pre-clearing steps employing non protein-conjugated Sepharose beads prior to the immunoprecipitation by FLAG antibody-conjugated beads. Lysates (input) and bead eluates (IP) were subjected to Western Blotting employing the indicated antibodies. SPPL2a and SPPL2b were detected with antibodies specific to the overexpressed murine proteins. The tetraspanins were either visualized with anti-FLAG or with specific antibodies directed against the respective proteins as indicated. Asterisk, antibody band. (J) Endogenous SPPL2b was precipitated from lysates of MEF WT cells (WT) and *SPPL2a/b*^*−/−*^ MEF cells (dKo) using anti-SPPL2b Sepharose. SPPL2b and CD9 were visualized using specific antibodies. Asterisk, antibody band.

We aimed to characterize the composition of the SPPL2a/b-containing complexes and tested an association of these proteases with tetraspanins in a candidate-based approach. CD9 and CD81 had been shown to associate with γ-secretase,^38^ which is mechanistically closely related to SPPL2a/b and shows an overlapping subcellular distribution.^1^ Furthermore, we also included CD82 as it was shown to form networks with CD9 and CD81.^47^^,^^48^^,^^49^ We co-expressed SPPL2a (Figures 2A–2C) or SPPL2b (Figures 2D–2F) with CD9 (Figures 2A and 2D), CD81 (Figures 2B and 2E), or CD82 (Figures 2C and 2F) in HEK293 cells. Following lysis with Brij-98, the respective tetraspanins were immunoprecipitated based on their fused 3xFLAG epitope. In all cases, the co-expressed protease was co-purified indicating presence in the same complex. When performing these co-IP experiments with CD9 and CD81, we also recovered the C-terminal fragment of endogenous presenilin 1 (PS1) (Figure S2A) along with co-expressed SPPL2a/b. We wanted to avoid the possibility of incomplete membrane solubilization which may result in unspecific co-immunoprecipitation of integral membrane proteins. Our concerns were dispelled by the fact that the endogenous epidermal growth factor receptor (EGFR) was not co-enriched when the respective tetraspanins were precipitated (Figures S2B–S2D). This confirms that the applied approach was capable of identifying tetraspanin-associated proteins.

We wanted to scrutinize the interactions under semi-endogenous conditions. Therefore, we stably transduced wild-type MEFs with FLAG-tagged CD9 (Figure 4A), CD81 (Figure 4B), and CD82 (Figure 4C) and performed pull-downs with anti-FLAG beads. Western blot analysis of the bead eluates confirmed enrichment of the tetraspanins. Endogenous SPPL2a and SPPL2b were co-precipitating with all three tetraspanins (Figures 4A–4C). As the EGFR could not be detected in MEFs, we probed for the transferrin receptor and the lysosomal protein TMEM192^50^^,^^51^ as negative controls for which no co-purification with the three tetraspanins was observed (Figures S2E–S2G). We attempted to repeat these co-immunoprecipitation experiments at the endogenous level. This was limited by performance and specificity of available antibodies in combination with low-endogenous protein levels. However, we achieved a pull down of endogenous SPPL2b from wild-type MEFs, where co-immunoprecipitation of CD9 was observed (Figure 2J). SPPL2a/b double-deficient cells were employed as negative control.

**Figure 3:**
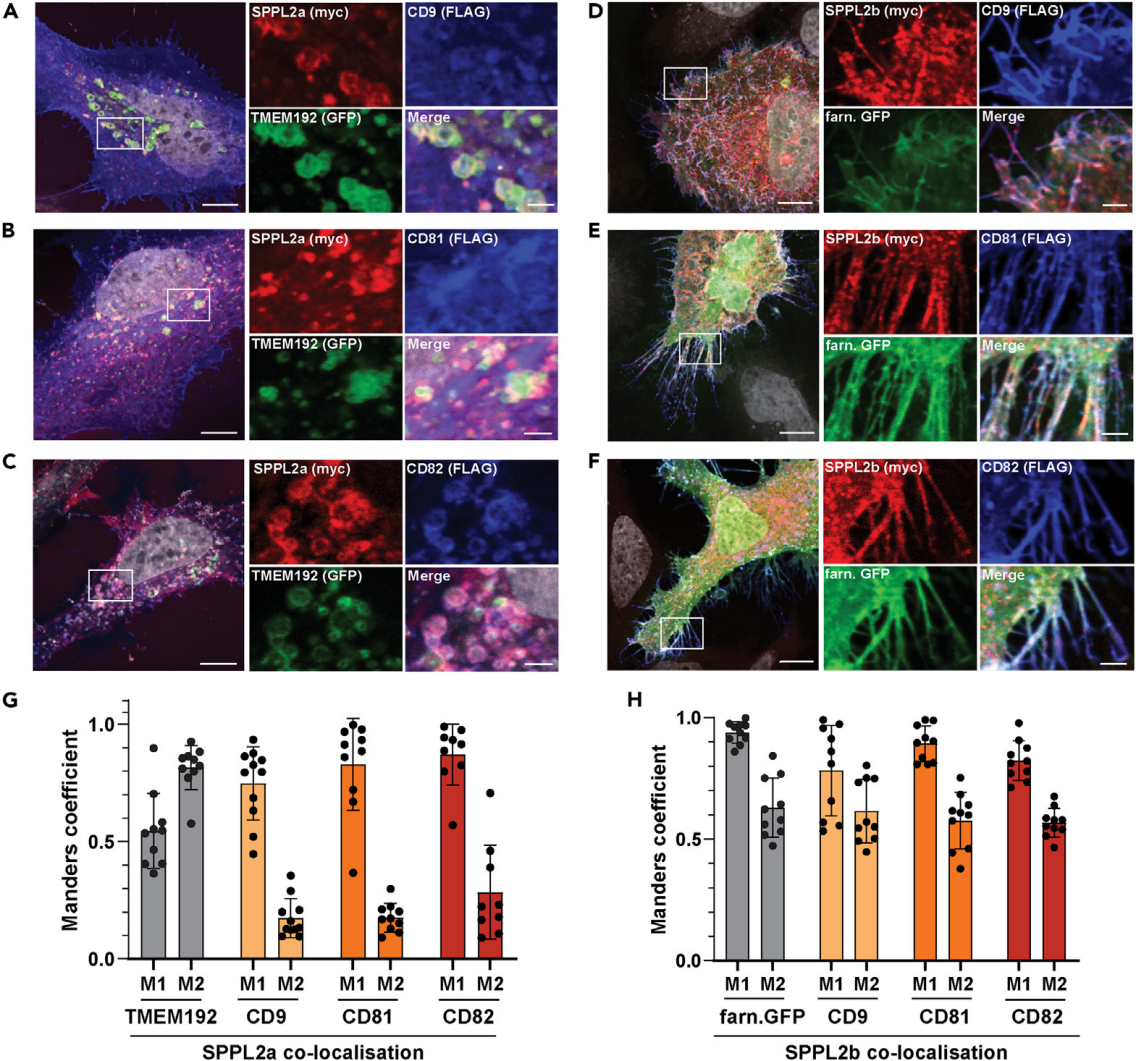
SPPL2a/b partially co-localize with CD9, CD81, and CD82 (A–F) Transient expression of SPPL2a (A–C) or SPPL2b (D–F) fused to a C-terminal Myc epitope in combination with CD9-3xFLAG (A and D), CD81-3xFLAG (B and E), or CD82-3xFLAG (C and F). In order to analyze the sub-cellular localization of SPPL2a and SPPL2b, the lysosomal protein TMEM192 fused to GFP and a construct expressing GFP fused to a farnesyl anchor, respectively, were co-expressed. After fixation, cells were subjected to indirect immunofluorescence analysis. SPPL2a/b and tetraspanins were visualized with anti-Myc or anti-FLAG, respectively, in combination with fluorophore-conjugated secondary antibodies. Visualization of the subcellular markers was based on GFP. Zoomed regions are represented by white boxes. Scale bars, 10 μm or 2 μm for the digital zoom. (G and H) Analysis of co-localization of SPPL2a (G) and SPPL2b (H) with the respective subcellular marker, CD9, CD81, and CD82 using Fiji and the JACop plugin. To estimate the proportion of SPPL2a/b either co-localizing with the respective analyzed tetraspanin or a subcellular marker, Manders co-localization coefficients (M1 and M2) were calculated by analysing individual cells. M1 is the percentage of above-background pixels in the image for the SPPL2a or SPPL2b staining that overlap with the above-background pixels in the image for the subcellular marker (TMEM192 or farnesylated GFP) or the analyzed tetraspanin. M2 provides information about the percentage of above-background pixels in the image for the staining of either the subcellular marker or the analyzed tetraspanins that overlap with the above-background pixels in the image for the SPPL2a or SPPL2b staining. Each data point represents one analyzed microscopic image. Mean ± S.D.

We analyzed the subcellular distributions of CD9, CD81, CD82, and SPPL2a/b upon overexpression in HeLa cells (Figure 3). In line with previous reports,^52^ SPPL2a (Figures 3A–3C) and SPPL2b (Figures 3D–3F) were predominantly present in vesicular, presumably lysosomal/endosomal compartments and at the plasma membrane, respectively. All three tetraspanins were prominently detected at the cell surface with varying amounts present in intracellular compartments, which was most prominent for CD82. We quantified co-localization of the two proteases with the three tetraspanins as well as TMEM192 (lysosomes) and a farnesylated variant of GFP (plasma membrane) as markers for the major reported localizations of SPPL2a/b (Figures 3G and 3H). Co-localization between CD9, CD81, CD82, and SPPL2b was very prominent at the plasma membrane, which was the predominant localization of these proteins (Figure 3H).

With regard to SPPL2a, the detailed images (Figures 3A–3C) as well as the co-localization quantification (Figure 3G, M1 coefficient) demonstrate that a large part of the SPPL2a-positive vesicular structures are also positive for CD9, CD81, or CD82 to a certain extent, although this is not the major localization of these proteins, which is demonstrated by the rather low-M2 coefficients. Nevertheless, the high-M1 coefficients indicate that SPPL2a has a significant overlap with these proteins and thus a chance to encounter CD9, CD81, and CD82 in the observed vesicular compartments, presumably belonging to the endolysosomal system. As not all SPPL2a-positive structures contained the lysosomal protein TMEM192, these may also include different types of endosomes. Altogether, the observed co-localizations of proteases and tetraspanins would be regarded as a prerequisite for being present in the same complex. The subcellular distribution of the proteases in cells co-expressing the three tetraspanins (Figure 3) was in line with their reported subcellular localization upon overexpression in HeLa cells.^52^ The localization of the tetraspanins in double-transfected cells did not differ from cells overexpressing only the tetraspanin (not shown). These findings argue against a direct impact of any SPPL2a/b-tetraspanin association on subcellular targeting.

### CD9 deficiency reduces DRM association of SPPL2b

**Figure 4:**
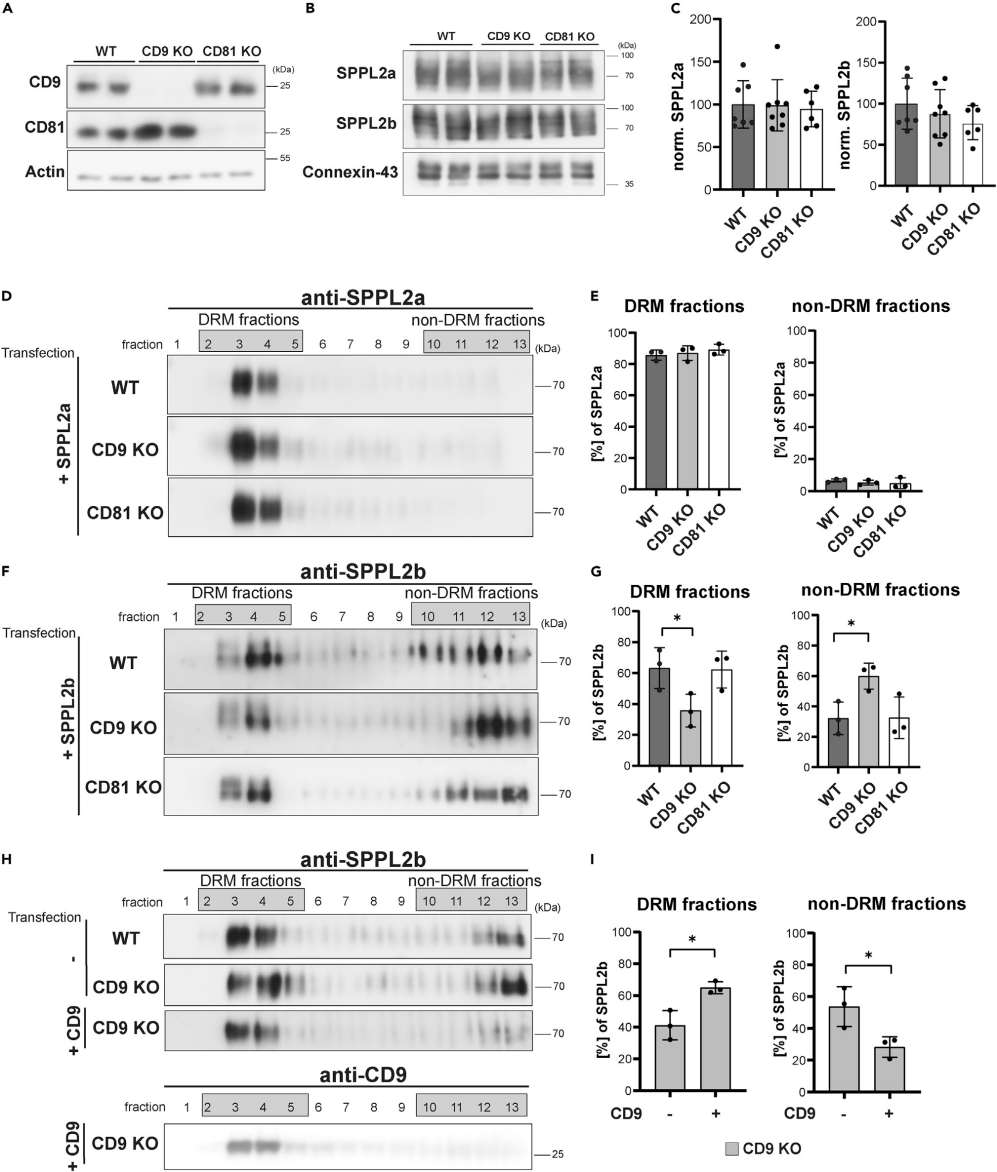
Deficiency of CD9 affects DRM-association of SPPL2b but not of SPPL2a (A) In order to validate the CD9 and CD81 deficiency, total lysates of wild type (WT), CD9 KO and CD81 KO HEK293 cells were analyzed by Western blot analysis using anti-CD9 and anti-CD81 antibodies. Actin was detected to confirm equal protein loading. (B) Total carbonate-washed membrane preparations from wild type, CD9 KO and CD81 KO HEK293 cells were analyzed on altered SPPL2a/b protein expression by Western blotting using anti-SPPL2a and anti-SPPL2b antibodies as well as detecting Connexin-43 as loading control. (C) Densitometric quantification of (A). Mean ± S.D., N = 3, n = 6–8. A one-way ANOVA with Tukey’s post-hoc test was performed. (D–G) HEK293 WT, CD9 KO, and CD81 KO cells were transiently transfected with SPPL2a (+SPPL2a, D and E) or SPPL2b (+SPPL2b, F and G) expression constructs to analyze the potentially altered association of the proteases in DRMs in cells lacking CD9 or CD81, respectively. After 24 h, cells were solubilized with 1% Brij-98 and lysates separated on discontinuous sucrose density gradients at 263,627 x g_max_ for 16 h at 4°C. Thirteen fractions were collected starting from the top and analyzed by Western blotting using antibodies against SPPL2a (D) or SPPL2b (F). (E) Quantification of (D). Band intensities of SPPL2a were densitometrically quantified in all fractions. Intensities within the DRM fractions (fractions 2–5) and non-DRM fractions (fractions 10–13) were combined, respectively, and are depicted as proportion (%) of the overall detected SPPL2a throughout the entire gradient. Mean ± S.D., N = 3, n = 3. (G) Quantification of (F). Distribution of SPPL2b in DRM and non-DRM fractions was quantified as described for SPPL2a in (E). Mean ± S.D., N = 3, n = 3. An unpaired, two-tailed Student’s t test was performed. ∗, p < 0.05. (H) HEK WT or HEK CD9 KO cells were transiently transfected with SPPL2b expression constructs either in combination with empty vector (−) with a CD9 expression plasmid (+CD9). Cell extracts were separated on discontinuous sucrose density gradients as described previously. Fractions were subjected to Western blot analysis to analyze the distribution of SPPL2b. CD9 was analyzed in parallel to confirm its re-expression in the CD9 KO cells. (I) Quantification of (H). The proportion of SPPL2b in DRM and non-DRM fractions was quantified as described for SPPL2a in (E). Mean ± S.D., N = 3, n = 3. An unpaired, two-tailed Student’s t test was performed. ∗, p < 0.05.

We aimed to analyze the functional relevance of the SPPL2a/b association with CD9, CD81, or CD82. Whereas CD82 does not seem to be expressed in HEK293 cells in relevant amounts (https://www.proteinatlas.org), endogenous CD9 and CD81 could be detected in this cell line while they were absent in the respective knockout cell lines (Figure 4A). Using these cell lines, we wanted to analyze how loss of these two tetraspanins may impact on properties and functions of SPPL2a and SPPL2b.

We determined steady-state levels of endogenous SPPL2a and SPPL2b in the knockout cells, which required enrichment of membrane proteins due to the limited detection sensitivity of the available antibodies (Figure 4B). No significant changes in total levels were seen in comparison to wild-type cells (Figure 4C). Furthermore, we assessed the subcellular localization of the overexpressed proteases (Figure S3) based on co-localization with the endogenous lysosomal marker LAMP2 and the co-expressed plasma membrane protein CADM1. The degree of co-localization with the respective markers (Figures S3G and S3H) was similar in all three cell lines. Based on this, we can conclude that in agreement with previous overexpression data (Figure 3), deficiency of CD9 or CD81 does not affect stability and subcellular targeting of SPPL2a/b.

We wondered if CD9 or CD81 deficiency would influence association of the two proteases with TEMs. As a correlate for this, we assessed their affinity to DRMs as in previous experiments (Figure 1). As Western blot detection of endogenous SPPL2a/b in the gradient fractions of HEK293 cells was unsuccessful with the available antibodies due to lack of sensitivity, we overexpressed the two proteases. Overexpressed SPPL2a was quantitatively recovered in the DRM fractions irrespective of whether wild type, CD9- or CD81-deficient cells were analyzed (Figures 4D and 4E). In wild-type cells, about two-thirds of overexpressed SPPL2b were present in DRMs (Figures 4F and 4G). Similar to the observations in MEFs and BMDCs, DRM association of SPPL2b in HEK cells was less complete than that of SPPL2a (Figures 4D and 4E). CD81 deficiency did not affect the distribution of SPPL2b between DRM and non-DRM fractions. However, in CD9-knockout cells the DRM-associated fraction of SPPL2b was reduced by about 50% of the level in wild-type cells demonstrating a re-distribution of SPPL2b within the gradient (Figures 4F and 4G). We also assessed a potential impact of CD9 and CD81 deficiency on DRM association of presenilin-1. However, the distribution of presenilin-1 was similar in gradient separations of Brij-98 extracts from the different cell lines (Figure S4).

**Figure 5:**
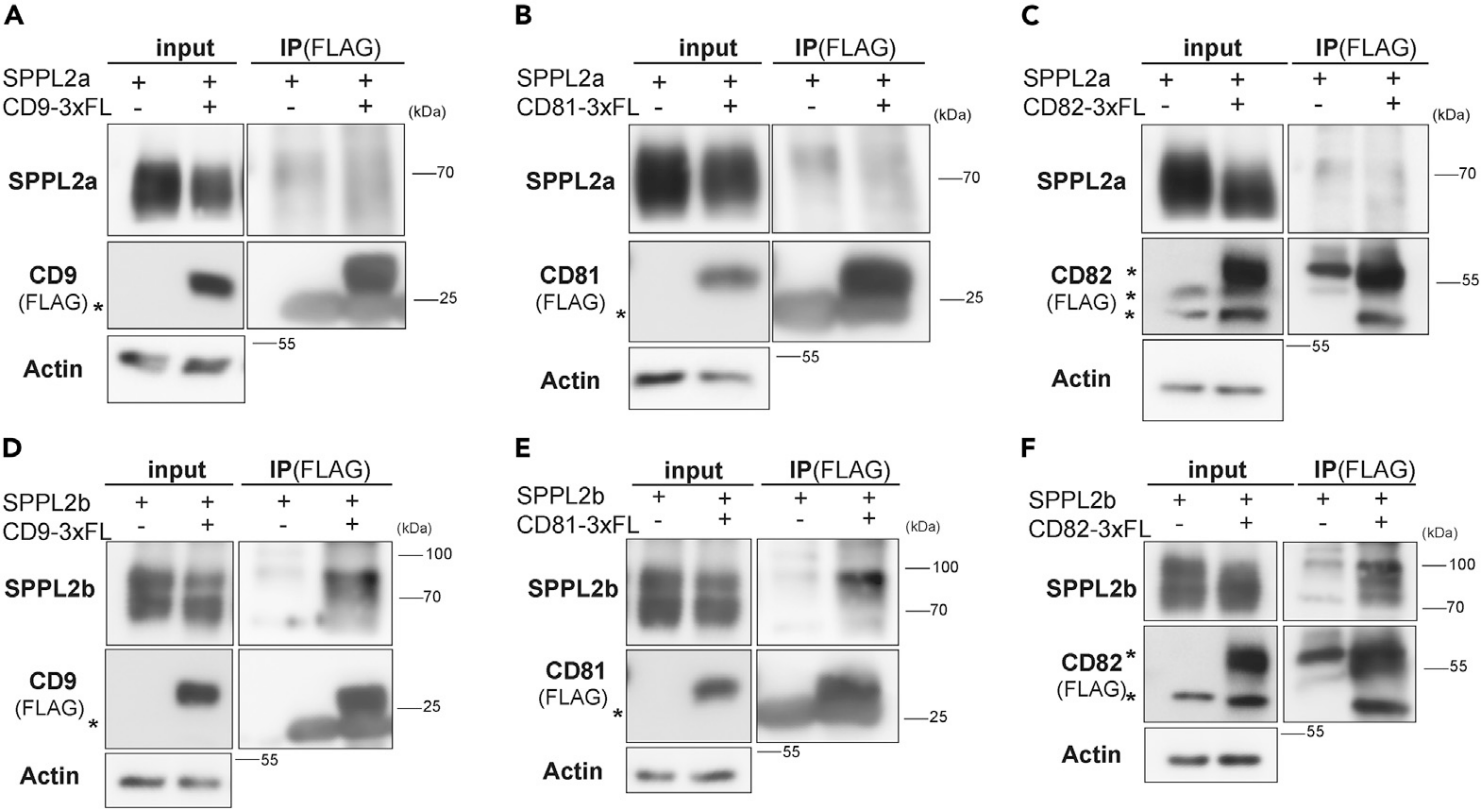
SPPL2b but not SPPL2a co-purifies with CD9, CD81, and CD82 following extraction with Triton X-100 (A–C) HEK293 cells were transiently transfected with either SPPL2a-Myc alone or in combination with CD9-3xFLAG (A), CD81-3xFLAG (B), and CD82-3xFLAG (C). (D–F) Similarly, SPPL2b-Myc alone or in combination with CD9-3xFLAG (D), CD81-3xFLAG (E), and CD82-3xFLAG (F) were transiently overexpressed in HEK293 cells. Cells were lysed with 1% Triton X-100 and triple FLAG-tagged (3x-FL) CD9, CD81, or CD82 were immunoprecipitated using FLAG antibody-conjugated beads. Lysates (input) and bead eluates (IP) were subjected to Western Blotting employing specific antibodies targeting murine SPPL2a or SPPL2b. The tetraspanins were visualized with anti-FLAG. Asterisk, antibody bands.

We wanted to further corroborate that the observed SPPL2b re-distribution was indeed caused by the CD9 deficiency. Therefore, we re-expressed CD9 in the CD9-knockout cells and analyzed the impact on the DRM association of SPPL2b (Figures 4H and 4I). Re-introduction of CD9 significantly increased the abundance of SPPL2b in the DRM fractions thereby restoring the distribution between DRM and non-DRM fractions to that of wild-type cells (Figures 4F and 4G). Altogether, this indicates that CD9 is involved in connecting SPPL2b to the tetraspanin network. On the contrary, TEM association of SPPL2a was not negatively affected in the analyzed set-up by loss of CD9 or CD81. These results demonstrate an impact of CD9 on SPPL2b, which was not shared by CD81 and not seen for SPPL2a. We wondered whether this may have a correlate at the level of the individual protease-tetraspanin complexes. As described, Triton X-100 dissolves TEMs as many interactions in these domains, both between different tetraspanin molecules but also with other membrane proteins, are rather weak. However, complexes of certain tetraspanins with specific partner proteins are preserved also in Triton X-100 indicating a hierarchy of protein-protein associations within TEMs.^29^^,^^30^^,^^53^^,^^54^ Based on this, we repeated the co-IP experiments as shown in Figures 2A–2F, however, extracted the cells with 1% Triton X-100 this time (Figure 5). Under these conditions, SPPL2a was not co-purified anymore with CD9 (Figure 5A), CD81 (Figure 5B), and CD82 (Figure 5C). However, SPPL2b was still co-enriched with all three tetraspanins (Figures 5D–5F). This indicates a higher stability of the respective SPPL2b-tetraspanin-containing complexes versus those formed by SPPL2a. Also in this experiment, the endogenous EGFR was probed as an unrelated negative control for the co-IP (Figure S5).

### The SPPL2a/b substrate TNF is also part of CD9 and CD81-containing complexes

**Figure 6:**
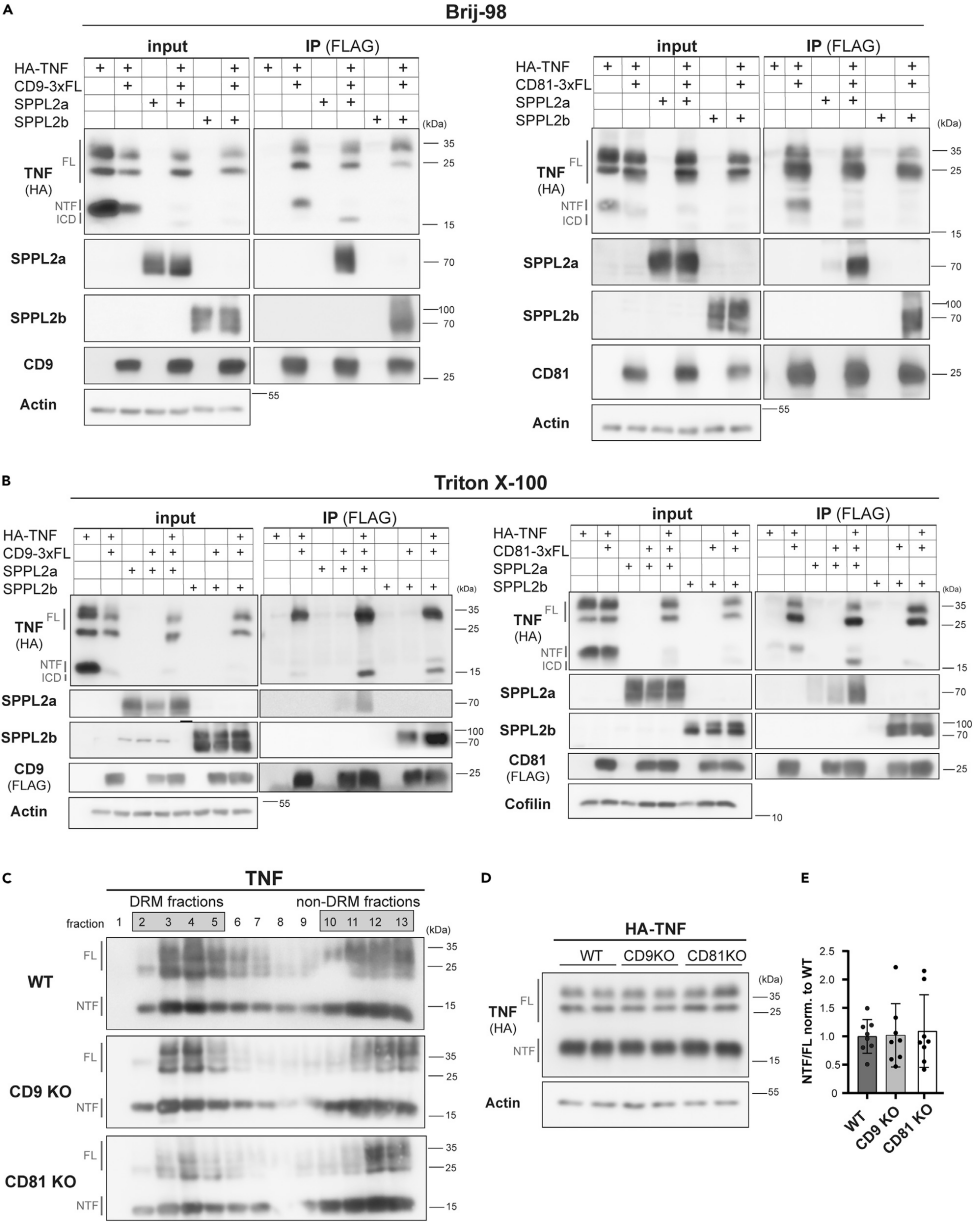
The SPPL2a/b substrate TNF forms complexes with tetraspanins HEK293 cells were transiently transfected as indicated in the figure with expression constructs for TNF with an N-terminally fused HA epitope (HA-TNF), SPPL2a-Myc, SPPL2b-Myc, CD9-3xFLAG, or CD81-3xFLAG. (A and B) Cells were lysed in either 1% Brij-98 (A) or 1% Triton X-100 (B), and pulldown of CD9 (left panel) or CD81 (right panel) was performed using FLAG antibody-conjugated beads. Total lysates (input) and beads eluates (IP) were analyzed by Western blotting employing anti-HA, specific SPPL2a and SPPL2b targeting antibodies, and anti-FLAG or antibodies directed against CD9 or CD81. Bands representing the full length form of TNF as well as the N-terminal fragment (NTF) resulting from ectodomain processing and the intracellular domain (ICD), the cleavage product generated by SPPL2a/b, are marked with “FL”, “NTF”, and “ICD”, respectively. (C) HEK293 WT, CD9 KO, and CD81 KO cells were transiently transfected with HA-TNF. Cells were extracted with 1% Brij-98 and lysates were separated on discontinuous sucrose density gradients at 263,627 x g_max_ for 16 h at 4°C. Thirteen fractions of 1 mL were collected from the top. Equal amounts of each fraction were analyzed by Western blotting using an antibody against the HA-tag to detect the full-length TNF (FL) and the TNF NTF (NTF). (D) Lysates of HEK293 WT, CD9 KO, and CD81 KO cells transiently expressing HA-TNF were subjected to Western blot analysis. HA-targeting antibody was used to detect the full-length TNF (FL) and the TNF NTF (NTF). Actin was detected to confirm equal protein loading. (E) Quantification of (D). Following densitometric quantification, levels of the TNF NTF were normalized to those of the full length protein (NTF/FL) and the resulting ratio was then normalized to that of the WT cells. Mean ± S.D., N = 4, n = 8. One-way ANOVA with Tukey’s post-hoc test was performed.

Having seen that loss of tetraspanins can affect the association of SPPL2 proteases with membrane domains, we wondered if this may affect substrate cleavage. As a prerequisite for this, it would need to be anticipated that substrates have access to the described protease-tetraspanin complexes. We evaluated this hypothesis for TNF^55^^,^^56^ as a prototypic SPPL2a/b substrate (Figure 6). When full-length, transmembrane TNF was expressed in HEK293 cells with an N-terminal HA epitope, bands representing the full-length protein (FL) as well as an NTF were detected. The latter results from the proteolytic release of the soluble cytokine and represents the actual SPPL2a/b substrate.^55^^,^^56^ Consequently, presence of active SPPL2a or SPPL2b depleted the NTF and produced a cleavage fragment comprising the intracellular domain (ICD). We co-expressed CD9 (Figures 6A and 6B left subpanel) or CD81 (Figures 6A and 6B right subpanel) as indicated. In analogy to experiments depicted in Figures 2 and 5, cells were lysed with 1% Brij-98 (Figure 6A) or 1% Triton X-100 (Figure 6B) and the tetraspanins recovered by pulldown. In samples with just the protease and the tetraspanin, but not the substrate TNF present, the recovery pattern of SPPL2a/b recapitulated results described previously (Figures 2 and 5). Thus, SPPL2a was only co-purified in Brij-98 extracts. TNF was co-enriched with both CD9 and CD81 and under both lysis conditions (Figures 6A and 6B). Both the full-length protein and the NTF were recovered. This indicates that—at least in this overexpression system—already the TNF precursor prior to any processing associates with CD9 and CD81 and suggests that generation of the NTF might take place in these complexes. An interesting observation was that upon co-expression of TNF, SPPL2a was co-immunoprecipitated with CD9 and CD81 also in presence of Triton X-100 (Figure 6B).

Based on the described association of TNF with CD9 and CD81, we analyzed its presence in DRMs as described previously (Figure 1). A significant fraction of TNF was detected in the top fractions of the gradient further supporting a connection to the tetraspanin network in this experimental system (Figure 6C). However, the degree of DRM association was not affected in the CD9 and CD81-deficient cell lines. Finally, we assessed if absence of CD9 or CD81 affects constitutive processing of TNF, in particular with regard to the turnover of the NTF by SPPL2a/b. Therefore, we determined steady-state NTF levels and quantified these in relation to the full-length protein (Figures 6D and 6E). We did not detect any indication of an alteration of TNF cleavage in the CD9- or CD81-knockout cells arguing against any general impairment of SPPL2a/b activity in these cell lines.

### CD81 deficiency impairs cleavage of the CD74 NTF by SPPL2a

**Figure 7:**
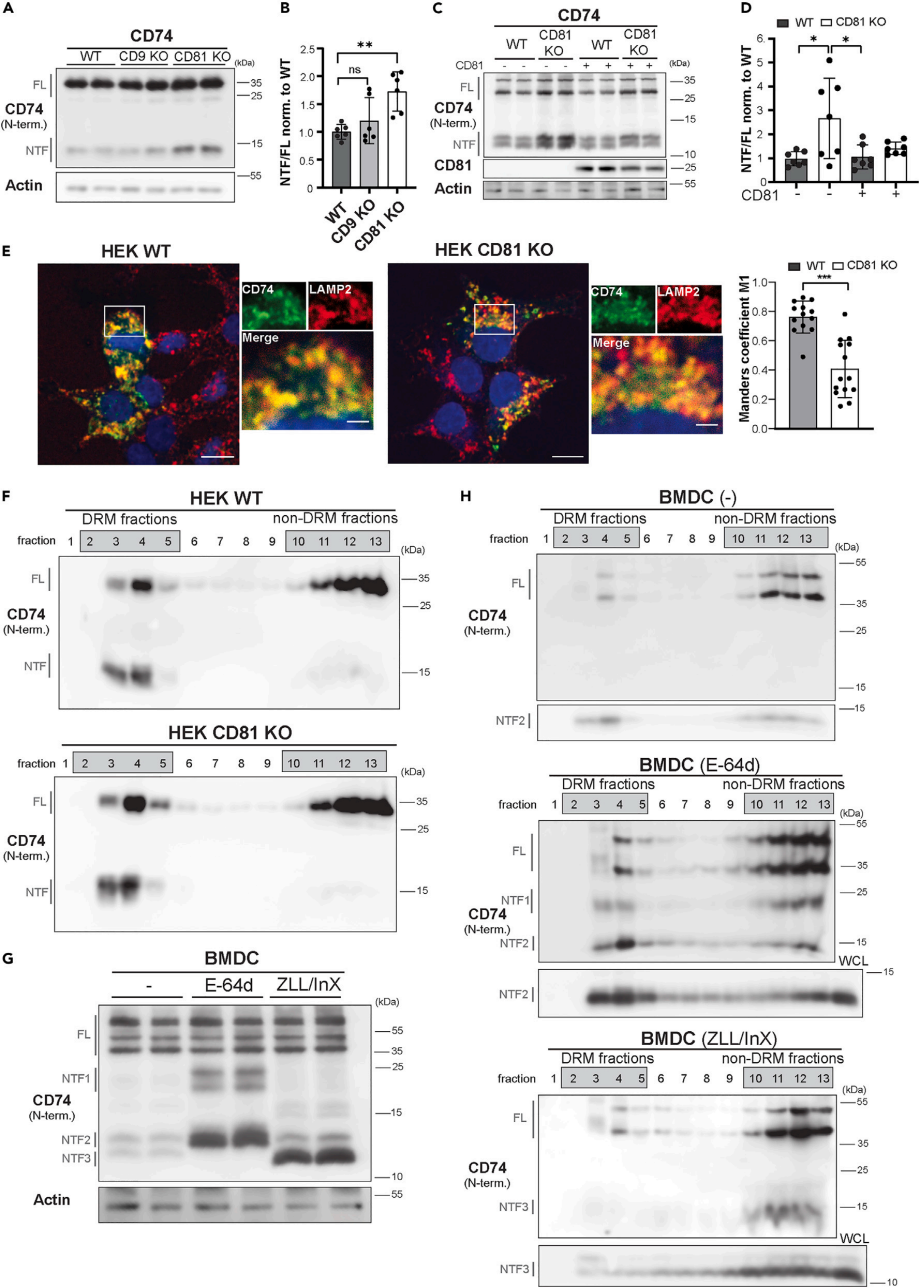
Intramembrane proteolysis of the CD74 NTF is impaired in CD81-deficient cells (A) CD74 was transiently expressed in HEK293 wild type (WT), CD9 KO, and CD81 KO cells. Total lysates were analyzed by Western blotting using a tris-glycine buffer system. CD74 was detected with an antibody against an N-terminal epitope detecting both the CD74 full length (FL) protein as well as derived N-terminal fragments (NTF). (B) Densitometric quantification of (A). Levels of the CD74 NTF were normalized to those of the full length protein (NTF/FL) and the resulting ratio then normalized to that of the WT cells. N = 3, n = 6. One-way ANOVA with Tukey’s post-hoc test. ∗∗, p < 0.01. (C) CD74 was transiently expressed in WT or CD81 KO HEK293 cells. As indicated, cells were co-transfected with a CD81-3xFLAG expression plasmid or empty vector (−). For detection of CD74, electrophoretic separation was performed with a tris-tricine buffer system which provides a better resolution of the individual forms and fragments of CD74. CD81 expression was also confirmed by Western blotting. (D–F) Quantification of (C). CD74 NTF/FL ratios were determined in the different samples and normalized to the level of the empty vector-transfected WT cells. Mean ± S.D. N = 4, n = 7. One-way ANOVA with Tukey’s post-hoc test. ∗, p < 0.05 (E) In order to analyze the subcellular targeting of CD74, wild type (WT) or CD81 KO HEK293 cells were transiently transfected with expression constructs of murine CD74. Following fixation, the distribution of CD74 was visualized using a polyclonal antiserum directed against the N-terminus of CD74 by indirect immunofluorescence. In parallel, the endo/lysosomal marker LAMP-2 was detected. Zoomed regions are represented by white boxes. Scale bar, 10μm or 2 μm for the digital zoom. The co-localization of CD74 and LAMP2 was analyzed using Fiji and the JACop plugin. Manders co-localization coefficient M1 was calculated which represents the percentage of above-background pixels in the images for the CD74 staining overlapping with the above-background pixels in the image for LAMP2. Each data point represents analysis of the CD74-expressing cells in one microscopic image. Mean ± S.D. N = 3, n = 13–14. One-way ANOVA with Tukey’s post-hoc test. ∗∗∗, p < 0.001 (F) CD74 was transiently expressed in WT and CD81 KO HEK293 cells. Lysates were prepared in presence of 1% Brij-98 and separated using discontinuous sucrose density gradients at 263,627 x g_max_ for 16 h at 4°C. Thirteen fractions of 1 mL were collected starting from the top. Equal volumes of each fraction were analyzed by Western blotting with an antibody detecting the N-terminus of CD74. (G) BMDCs from wild-type mice were treated with the cysteine protease inhibitor E−64d (40 μM), a combination of (Z-LL)_2_-ketone (40 μM) and inhibitor X for (1 μM) (ZLL/InX) for inhibiting SPPL2a or DMSO as control (−) for 24 h prior to cell harvest. Lysates were subjected to Western blotting employing a tris-tricine buffer system in order to analyze processing of endogenous CD74. Different NTFs were detected depending on the applied inhibitors which are referred to as NTF1, NTF2, and NTF3 in the following. (H) BMDCs treated as described in (G) were lysed in presence of 1% Brij-98. Lysates were separated by density gradient centrifugation as in (F). Fractions were analyzed by Western blotting (tris-glycine buffer system) for presence of full length CD74 and the different NTFs. To provide better separation of the distinct NTFs, samples were analyzed in parallel by tris-tricine SDS-PAGE prior to Western blot analysis, which is shown below the tris-glycine blots. In order to allow identification of the different NTFs by correlation with (G), total lysates of BMDCs (WCL) treated with the same inhibitors were analyzed in parallel to the respective gradient fractions when performing the tris-tricine SDS-PAGE.

We sought to analyze the processing of another substrate and evaluated proteolysis of CD74 (Figure 7), which can be cleaved by both SPPL2a and SPPL2b upon overexpression, while SPPL2a has a leading role in this *in vivo*^10^ Again the steady-state abundance of the NTF, which is the actual substrate of SPPL2a/b,^9^^,^^10^ was assessed and compared between the different cell lines after normalization to that of the full-length precursor (Figures 7A and 7B). Whereas the CD74 NTF/FL ratio in the CD9-knockout cells was similar to that of the control cells, CD81-deficient cells exhibited a significant NTF accumulation. We wanted to confirm that this effect is specifically linked to the loss of CD81. CD81 expression in the knockout cells by transient re-transfection completely reversed the CD74 NTF accumulation to the level of wild-type cells (Figures 7C and 7D). Altogether, this indicates that CD81 deficiency impairs cleavage and turnover of the CD74 NTF by SPPL2a/b, whereas this is not affected by loss of CD9.

We analyzed the targeting of CD74 as a potential reason for this altered processing (Figure 7E). In both wild type and CD81-knockout cells, CD74 prominently colocalized with the lysosomal protein LAMP2, though according to the performed quantification, the overlap was reduced in the CD81 KO cells. As lysosomal proteases are involved in processing the CD74 ectodomain and generating the NTF,^57^ a slightly reduced targeting to lysosomal compartments would not be able to explain an increased NTF abundance in these cells.

### Differential TEM association of CD74 during proteolytic processing

We tested if overexpressed CD74 is associated with DRMs as a correlate for TEMs in HEK WT cells (Figure 7F, top). Only about ∼30% of the full-length protein was detected in the DRM fractions. However, the observed NTF representing an intermediate of the sequential degradation was rather quantitatively recovered in the floating fractions. We considered that loss of CD81 may affect the presence of CD74 or its NTF in DRMs. However, there was no obvious difference in the distributions in the gradient fractions between wild type and CD81-deficient cells (Figure 7F). Thus, despite impairing cleavage of the CD74 NTF loss of CD81 does not affect its connection to DRMs on a global level.

We were intrigued by the prominent DRM localization of the CD74 NTF and wanted to scrutinize our observations in BMDCs, which express CD74 at the endogenous level as well as MHCII as its physiological-binding partner. Under steady-state conditions, mainly full length CD74 but rather little NTF was detected indicating that the different degradation steps occur in a highly synchronized manner (Figure 7G). We interfered with different steps of CD74 degradation by applying E−64d or a combination of (Z-LL)_2_-ketone and inhibitor X (ZLL/InX) in order to inhibit lysosomal cysteine proteases or SPPL2a/b, respectively. E−64d blocked the stepwise degradation of the CD74 luminal domain and stabilized different NTFs (NTF1 and NTF2) with apparent molecular weights of ∼20 and 10 kDa. NTF2 is further processed to NTF3, which represents the actual SPPL2a substrate and accumulates upon SPPL2a/b inhibition (Figure 7G).

As compared to the HEK cells, the DRM-associated fraction of full-length CD74 in BMDCs was low under steady-state conditions being in a range of ∼10% of the total pool (Figure 7H, top). NTF1 was similarly distributed in the gradient fractions as full-length CD74. However, NTF2 was very strongly enriched in the DRM fractions, where also SPPL2a was recovered, which reflects the observations from HEK293 cells (Figure 7H). This confirms at the endogenous level that during ectodomain processing of CD74, the affinity for DRMs/TEMs significantly increases. This occurs shortly before the intramembrane cleavage is supposed to take place. Surprisingly, the further trimmed NTF3 has lost the DRM/TEM affinity. It was primarily detected in the non-DRM fractions after the gradient separation. Nevertheless, these findings suggest that the step of CD74 processing, which produces the actual SPPL2a substrate, occurs in DRMs/TEMs and thus in proximity to the intramembrane protease.

**Figure 8:**
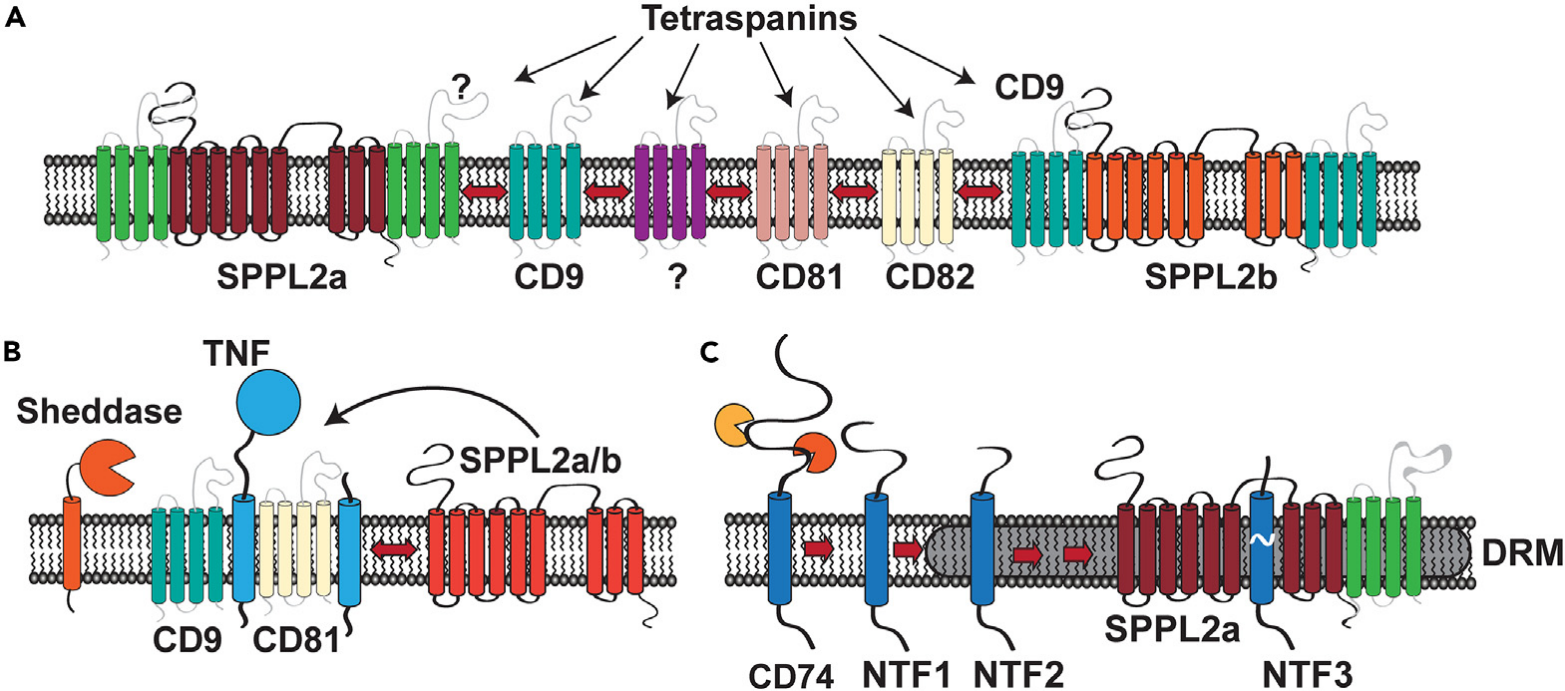
Proposed model for the impact of tetraspanins on SPPL2a/b (A) The presented findings indicate that both intramembrane proteases SPPL2a and SPPL2b are connected to tetraspanin-enriched domains (TEMs). Co-IPs in different detergents suggest a hierarchy of protease-tetraspanin interactions. Complexes between SPPL2b to CD9, CD81, and CD82 seem to be more stable upon detergent extraction than between SPPL2a and the three tetraspanins. As loss of CD9 reduced association of SPPL2b with DRMs/TEMs, it may be speculated that CD9 is a primary interaction partner of SPPL2b connecting this protease to the tetraspanin network. However, this will need to be corroborated by independent approaches. It is unclear if a similar tetraspanin interaction partner of SPPL2a forming a Triton X-100-stable complex exists. If so, this remains to be identified. Based on our co-immunoprecipitation experiments following Brij-98 extraction, we can conclude that both proteases have the propensity to connect to a variety of tetraspanins including but presumably not limited to CD9, CD81, and CD82. (B) Substrates of SPPL2a/b also can associate with tetraspanins. Both, the TNF full-length protein as well as N-terminal fragments after removal of the ectodomain were recovered in CD9- and CD81-containing complexes, which were stable even in Triton X-100. Presence of TNF also enhanced the complex formation between SPPL2a/b and CD9 or CD81. (C) Full-length CD74 was associated with Brij-98-resistant membranes/TEMs only to a minor degree. However, an NTF arising during ectodomain processing (NTF2) was very efficiently recruited to such complexes. Despite the described differences regarding the association of CD74 or TNF with tetraspanins, this could suggest a model that reciprocal associations of the transmembrane substrate and the intramembrane protease with tetraspanins assist in bringing these two partners into contact within the plane of the membrane. Loss of CD81 impaired proteolysis of CD74 NTFs but did not affect processing of TNF. This may suggest that distinct substrate-tetraspanin-protease complexes exist, which then facilitate individual cleavage events. The precise composition of these complexes remains to be determined.

We have observed that both the intramembrane proteases SPPL2a/b (Figure 8A) as well as their substrates (Figures 8B and 8C) can associate with different tetraspanins and/or DRMs with characteristics of TEMs. Our results suggest a certain hierarchy among the described SPPL2a/b-tetraspanin complexes as discussed below (Figure 8A). Altogether we would like to propose a model that reciprocal and dynamic associations with tetraspanins may help to facilitate proteolytic processing by the intramembrane proteases SPPL2a/b.

## Discussion

Our results add SPPL2a/b to the list of tetraspanin-associated membrane-embedded proteases. We showed that loss of CD9 significantly affected TEM association of SPPL2b but not of SPPL2a, whereas loss of CD81 reduced proteolysis of CD74 but not of TNF. These effects were thus restricted to a particular protease or a particular substrate. Though being rather subtle, they support that the association with tetraspanins is of functional relevance for SPPL2a/b. The precise layout of any protease-tetraspanin complexes currently remains elusive. In general, TEMs were initially proposed to contain different tetraspanins and partner proteins.^30^^,^^49^ Accordingly, CD9, CD63, CD81, and CD82 were detected in TEMs, in which several tetraspanins overlapped at least partially.^47^ On the contrary, individual nano-clusters of CD37, CD53, CD81, and CD82 were observed using super-resolution microscopy.^32^ In line, interactions of tetraspanins were found to be largely homotypic with parts of the large extracellular loop mediating homodimerization thereby initiating TEM formation,^58^^,^^59^ while other TEM-associating proteins were present both inside TEMs or at their edge.^32^^,^^60^ The co-IPs performed here, while demonstrating presence in a shared complex, are unable to prove a direct interaction between SPPL2a/b and the three evaluated tetraspanins. Our results suggest a certain hierarchy regarding stability among the analyzed tetraspanin-protease complexes. The association of SPPL2b with CD9, CD81, and CD82 was stable in the presence of Triton X-100, while SPPL2a-containing complexes were not. It was proposed that proteins connecting to TEMs may undergo certain primary interactions with a specific tetraspanin, which often were found to be stable in more stringent non-ionic detergents like Triton X-100.^29^^,^^30^^,^^53^^,^^54^ These primary complexes may then connect to other complexes and further tetraspanins by more labile secondary interactions which are only stable in Brij-type detergents.^29^^,^^30^^,^^53^^,^^54^ With all caution, one could speculate that the association of SPPL2b with CD9 falls into the category of a primary interaction based on its Triton X-100 stability and the reduced DRM/TEM association in CD9-knockout cells as visualized in our model in Figure 8A. On the other hand, none of the three examined tetraspanins may represent a primary partner of SPPL2a but may rather be connected to this protease by secondary interactions. This would then imply that any primary partner connecting SPPL2a to the tetraspanin network remains to be identified. More direct approaches will be required in the future for characterizing proximity of the respective partners in SPPL2a/b-tetraspanin complexes to underpin this currently rather indirectly supported model (Figure 8A). As revealed by single molecule-tracking analysis of CD9, interactions within the tetraspanin web^60^ are highly dynamic, which will make such an analysis even more complex.

We have demonstrated that also the SPPL2a/b substrates TNF (Figure 8B) and CD74 (Figure 8C) have an affinity for tetraspanins and/or DRMs/TEMs. We were able to co-immunoprecipitate TNF with CD9 and CD81 even following Triton X-100 extraction (Figure 8B). Previously, Tspan8 (CO-029) was found to associate with TNF.^61^ Interestingly, Tspan8 belongs to the same tetraspanin subfamily as CD9 and CD81.^62^ Tspan8 promoted cleavage of TNF but not of other substrates by ADAM17^61^ thus affecting proteolysis in a substrate-selective way. CD74 (and also MHCII) was previously detected in Brij-98 DRMs, while no flotation following CHAPS or Triton X-100 extraction was observed.^63^ Both CD9 and CD81 have been reported to form complexes with MHCII,^27^^,^^48^^,^^49^ which is bound by CD74 in the ER.^57^ Steps of the CD74 ectodomain processing may differ slightly between overexpressing HEK293 cells and BMDCs due to the absence of MHCII in the first and variations in the expressed cathepsins. Nevertheless, in both systems DRM/TEM recruitment was seen only for a specific degradation intermediate (Figure 8C). This is independent of an association with MHCII as it was also seen in HEK293 cells and instead represents an intrinsic property of this CD74 NTF. As the shorter NTF3 in BMDCs has lost DRM affinity, one could speculate that a specific part of the luminal domain plays a role here. However, it remains to be clarified why full-length CD74 does not show this property. CD74 contains a palmitoylation site,^64^ which may be dynamically utilized and may also play a role in this context.^23^ Loss of CD81 had certain effects on CD74 trafficking and impaired turnover of the NTF by SPPL2a. Nevertheless, DRM association of the CD74 NTF was not altered in the CD81-knockout cells suggesting an involvement of further tetraspanins.

The observed tetraspanin association of SPPL2a/b and their substrates suggests a model that tetraspanins with their general propensity to interact and to build networks could facilitate protease-substrate encounters (Figures 8B and 8C) in the membrane. The observation that SPPL2a was recovered in a Triton X-100 stable complex with CD9 and CD81 only when the substrate TNF was present could, with all caution, indicate recruitment of the protease into pre-existing TNF-tetraspanin complexes. Again, further analysis will be required to substantiate this model.

Cellular membranes are heavily crowded with protein contents of ∼50% and observed area occupancies in the range of 25%,^65^ which restricts and slows down lateral diffusion of membrane proteins.^66^ This poses a challenge to membrane-embedded proteolytic processes, in particular when several cleavage events by different proteases are coupled in an RIP sequence.^3^ In this context, mechanisms bringing together membrane-bound proteases and their substrates, e.g., based on tetraspanins, seem to be a plausible concept, in particular, as intramembrane proteases are considered to be intrinsically slow enzymes.^67^ This model has been elaborated for processing of the amyloid precursor protein (APP) by demonstrating an association between ADAM10 and γ-secretase, in which the tetraspanins Tspan12 and Tspan17 were involved.^68^ Tetraspanin interactions of ectodomain-shedding proteases have been analyzed broadly in the case of ADAM10,^39^^,^^69^^,^^70^^,^^71^^,^^72^^,^^73^^,^^74^ ADAM17,^61^^,^^75^^,^^76^ and meprin β.^77^ SPPL2a/b functions in RIP sequences initiated by ADAM17 or ADAM10, as e.g., in the processing of TNF^55^^,^^56^ or Bri2,^78^ respectively.

With regard to other intramembrane proteases, our findings on SPPL2a/b show a striking resemblance to a previous report on γ-secretase, which was associated with DRMs isolated in presence of Brij-99 but not Triton X-100.^38^ Both CD9 and CD81 could be co-immunoprecipitated with presenilins and the other accessory subunits of the γ-secretase complex.^38^ CD9-, and much more subtly, CD81-deficient MEFs displayed a partial disruption of γ-secretase activity demonstrated by a significant accumulation of C-terminal fragments of endogenous substrates.^38^ For other members of the SPP/SPPL family than SPPL2a/b and other non-aspartic intramembrane proteases, like site-2 protease or rhomboids, a DRM or tetraspanin association has not been reported to our knowledge. Based on previous concepts, our findings provide further support that the capability of tetraspanins proteins to segregate membrane proteins and organize membranes is important for membrane-embedded proteases. This strongly advocates to evaluate this also for the remaining intramembrane proteases and to obtain more mechanistic insight into how the respective complexes are organized.

### Limitations of the study

Proof of association of SPPL2 proteases and/or their substrates was obtained in co-immunoprecipitation experiments. Due to the limited availability of functional antibodies, only some of the discovered protein associations could be scrutinized at the full endogenous level, while at least one of the associating proteins had to be overexpressed in relevant parts of the experiments. In general, co-immunoprecipitation analysis requires integral membrane proteins to be solubilized with detergents in order to disrupt the phospholipid bilayer. However, studying membrane protein complexes following solubilization, thus after their release from their natural environment, bears a certain intrinsic risk of false-negative or false-positive identification of protein interactions. Therefore, approaches studying complex formation *in situ* will be important in the future to complement our data, which, however, have other limitations. These may also help to generate insights into how direct or indirect the associations of proteases and tetraspanins in the complexes are which we describe here based on co-immunoprecipitation. A general challenge when working with tetraspanins is this protein family’s diversity and high degree of functional redundancy. Based on this, depletion of single tetraspanins, as also seen in this study, often has rather limited effects.

## STAR★Methods

### Key resources table

**Table:** 

REAGENT or RESOURCE	SOURCE	IDENTIFIER
**Antibodies**		

monoclonal anti-mouse CD9 (clone KMC8)	BD Biosciences	Cat# 553758, RRID:AB_395032
monoclonal anti-human CD9 (clone D3H4P)	Cell Signaling Technology	Cat# 13403, RRID:AB_2732848
monoclonal anti-mouse CD81 (clone D5O2Q)	Cell Signaling Technology	Cat# 10037, RRID:AB_2714207
monoclonal anti-human-CD81 (clone E2K9V)	Cell Signaling Technology	Cat# 52892, RRID:AB_2924773
monoclonal anti-Presenilin 1 (clone D39D1)	Cell Signaling Technology	Cat# 5643, RRID:AB_10706356
monoclonal anti-Cofilin (clone D3F9)	Cell Signaling Technology	Cat# 5175, RRID:AB_10622000
monoclonal anti-FLAG (clone M2)	Sigma-Aldrich	Cat# F3165, RRID:AB_259529
monoclonal anti-HA (clone 3F10)	Roche	Cat# 11867423001, RRID:AB_390918
monoclonal anti-myc (clone 9B11)	Cell Signaling Technology	Cat# 2276, RRID:AB_331783
monoclonal anti-LAMP2 (clone 2D5)	Radons et al.^79^	N/A
polyclonal anti-Transferrin receptor (clone ab84036)	Abcam	Cat# ab84036, RRID:AB_10673794
monoclonal anti-Transferrin receptor (clone H68.4)	Cell Signaling Technology	Cat# 46222
polyclonal anti-Actin (clone A2066)	Sigma-Aldrich	Cat# A2066, RRID:AB_476693
polyclonal anti-Connexin 43 (clone 3512S)	Cell Signaling Technology	Cat# 3512, RRID:AB_2294590
monoclonal anti-EGFR (clone D38B1)	Cell Signaling Technology	Cat# 4267
monoclonal anti-Flotillin 1	BD Biosciences	Cat# 610820, RRID:AB_398139
polyclonal anti-α-tubulin	Cell Signaling Technology	Cat# 2144, RRID:AB_2210548
polyclonal anti-TMEM192 (murine)	Nguyen et al.^80^	N/A
monoclonal CD74 (clone In-1)	BD Biosciences	Cat# 555317, RRID:AB_395727
polyclonal anti-mouse SPPL2a	Behnke et al.^52^	N/A
polyclonal anti-human SPPL2a	Schneppenheim et al.^81^	N/A
polyclonal anti-mouse SPPL2b	Schneppenheim et al.^10^	N/A
polyclonal anti-human SPPL2b	Mentrup et al.^11^	N/A
polyclonal anti-mouse CD74	This paper	N/A

**Bacterial and virus strains**		

*E. coli* XL1- Blue	Agilent	Cat# 200249
*E. coli* CopyCutter™ EPI400™ Electrocompetent strain	Epicentre	

**Chemicals, peptides, and recombinant proteins**		

(Z-LL)_2_-ketone	Peptanova	Cat# 3218-v
1,4-diazabicyclo[2.2.2]octane (DABCO)	Sigma-Aldrich	Cat# D27802
4′,6-Diamidino-2-phenylindole dihydrochloride (DAPI)	Sigma-Aldrich	Cat# D9542
Anti-FLAG M2 agarose gel	Sigma-Aldrich	Cat# A2220
Complete Protease Inhibitor Cocktail	Roche	Cat# 11697498001
CopyCutter Induction Solution™	Epicentre	Cat# 191315
Dulbecco’s modified Eagle’s medium (DMEM)	Gibco	Cat# 11594486
E-64d	Enzo Lifesciences	BML-PI107-0001
ECL Western Blotting Detection Substrate	Bioquote	Cat# B18001
Fetal calf serum (FCS)	Thermo Fisher Scientific	
Inhibitor X	Tocris	Cat# 2627
Mowiol 4-88	Sigma-Aldrich	Cat# 81381
Pefabloc® SC Protease Inhibitor	Carl Roth	Cat# A154.1
PEI MAX Transfection Grade Linear Polyethylene	Polysciences	Cat# 24765-1
Pepstatin A	Sigma-Aldrich	Cat# P5318
Polybrene	Sigma-Aldrich	Cat# TR-1003-G
Recombinant GM-CSF	Immunotools	Cat# 12343122
RPMI 1640	Gibco	Cat# 11875093
Saponine	Carl Roth	Cat# 9622.1

**Critical commercial assays**		

Nucleospin RNA Plus extraction kit	Macherey-Nagel	Cat# 740984.50
Pierce BCA Protein Assay Kit	Thermo Fisher Scientific	Cat# 23227
Pierce™ Direct IP Kit	Thermo Fisher Scientific	Cat# 26148
RevertAid First Strand cDNA Synthesis Kit	Thermo Fisher Scientific	Cat# K1621

**Experimental models: Cell lines**		

Human: HEK293T	DSMZ	RRID:CVCL_0063
Human: HEK293T CD9 KO	T. Gallagher (Loyola University, Chicago), Earnest et al.^82^	N/A
Human: HEK293T CD81 KO	T. Gallagher (Loyola University, Chicago), Earnest et al.^82^	N/A
Human: HeLa	DSMZ	RRID: CVCL_0030
Human: Platinum-E	Cell Biolabs Inc.	RRID:CVCL_B488
Mouse: immortalized MEF	Mentrup et al.^11^	N/A
Mouse: SPPL2a/b^-/-^ MEF	Mentrup et al.^11^	N/A
Mouse: primary BMDC	This paper	N/A

**Experimental models: Organisms/strains**		

Mouse: Wild type C57BL/6 N *Crl*	Own breeding	N/A

**Oligonucleotides**		

mCD82-BgllI forward primer GATCAGATCTGCCACCATGGGGG CAGGCTGTGTCAAAGTC	This paper	N/A
mCD82-XhoI-3xFLAG reverse primer GATCCTCGAGTCACTTGTCGTCGTCGTCC TTGTAGTCGATGTCGTGGTCCTTGTAG TCGCCGTCGTGGTCCTTGTAGTCGTAC TTGGGGACCTTGCTGTAG	This paper	N/A
mCD81-BgllI forward primer GATCAGATCTGCCACCATGGGGGTGG AGGGCTGCACCAAAT	This paper	N/A
mCD81- XhoI-3xFLAG reverse primer GATCCTCGAGTCACTTGTCGTCGTCGTCC TTGTAGTCGATGTCGTGGTCCTTGTAG TCGCCGTCGTGGTCCTTGTAGTCG TACACGGAGCTGTTCCGGAT	This paper	N/A
mCD9-BgllI forward primer GATCAGATCTGCCACCATGCCGGTC AAAGGAGGTAGCAAG	This paper	N/A
mCD9-XhoI-3xFLAG reverse primer GATCCTCGAGCTACTTGTCGTCGTCGTCC TTGTAGTCGATGTCGTGGTCCTTGTAG TCGCCGTCGTGGTCCTTGTAGTCGACCA TTTCTCGGCTCCTGCGGA	This paper	N/A
mCD74-BamHI forward primer GATCGGATCCACGCCACCATGGATG ACCAACGCGACCT	This paper	N/A
mCD74-XhoI reverse primer GATCCTCGAGTCACAGGGTGACTTGA CCCAGTTC	This paper	N/A
mCADM1-HindIII forward primer GATCAAGCTTACGCCACCATGGCG AGTGCTGTGCTGCCGAG	This paper	N/A
mCADM1-NotI reverse primer GATCGCGGCCGCCTAAGCGTAGTC TGGGACGTCGTATGGGTAGATGAA GTACTCTTTCTTTT	This paper	N/A
GFP-HindIII forward primer TCAAAGCTTGCCACCATGGTGAGCAAG GGCGAGGAGCTGTTC	This paper	N/A
GFP-Farnesylated XhoI reverse primer GCAC TCGAGTCAGGAGAGCACACACTTGCAGC TCATGCAGCCGGGGCCACTCTCA TCAGGAGGGTTCAGCTTCTTGTACAGC TCGTCCATGCCG	This paper	N/A
hTMEM192 forward primer CTCGAGCTCAAGCTTCGACAAGTTTGTA CAAAA	This paper	N/A
hTMEM192 reverse primer TGGATCCCGGGCCCGCGTTCTACTT GGCTG	This paper	N/A

**Recombinant DNA**		

Plasmid: pcDNA3.1 hygro(+) mSPPL2a-myc	Behnke et al.^52^	N/A
Plasmid: pcDNA3.1 hygro(+) mSPPL2b-myc	Behnke et al.^52^	N/A
Plasmid: pcDNA3.1 hygro(+) mCD9-3xFLAG	This paper	NM_007657.4
Plasmid: pcDNA3.1 hygro(+) mCD81-3xFLAG	This paper	NM_133655.2
Plasmid: pcDNA3.1 hygro(+) mCD82-3xFLAG	This paper	NM_001136055.2
Plasmid:pMSCV puro mCD9-3xFLAG	This paper	NM_007657.4
Plasmid:pMSCV puro mCD81-3xFLAG	This paper	NM_133655.2
Plasmid:pMSCV puro mCD82-3xFLAG	This paper	NM_001136055.2
Plasmid: pcDNA3.1 hygro(+) HA-mTNF-V5	Mentrup et al.^83^	N/A
Plasmid: pcDNA3.1 hygro(+) mCD74 p31	This paper	N/A
Plasmid: pcDNA3.1 hygro(+) mCADM1-HA	This paper	N/A
Plasmid:p-eGFP-N1 hTMEM192	This paper	N/A
Plasmid: pcDNA3.1 hygro(+) farnesylated GFP	This paper	N/A
Software and algorithms		
GNU Image Manipulation Program software (version 2.10.14)	https://www.gimp.org/	RRID:SCR_003182
ImageJ (version 1.52a)	https://imagej.nih.gov/	RRID:SCR_003070
GraphPad Prism (version 8.4.3)	GraphPad Prism Software	RRID:SCR_002798

### Resource availability

#### Lead contact

For additional information and inquiries regarding resources and reagents, please contact Bernd Schröder (bernd.schroeder@tu-dresden.de).

#### Materials availability

Materials generated in this study will be available upon request.

### Experimental model and study participant details

#### Animals

Wild type C57BL/6 N Crl mice were initially obtained from Charles River and then bred further. Mice were kept at a temperature of 19°C-21°C, a relative humidity of 45-60% and a circadian rhythm of 12 h lightness and 12 h darkness. Animal experimentation was performed in agreement with national and local guidelines for the use of animals and their care and had been approved by the Landesdirektion Sachsen (TV A 12/2018, DD24.1-5131/450/12). Experiments were performed with mice at an age of 12-14 weeks. Both male and female mice were used, as sex did not influence outcome of the performed experiments.

#### *E. coli* strains

Two different electro-competent strains of *E. coli* were utilized to propagate plasmids. The CopyCutter™ EPI400™ Electrocompetent strain (Epicentre), optimized for amplifying unstable or toxic DNA with low copy numbers, was used to propagate vectors carrying SPPL2a or its catalytically inactive mutant. Four hours prior to cell harvest, the generation of high copy numbers plasmids was induced by treating the bacteria with CopyCutter Induction Solution™ (Epicentre) at a 1:1000 ratio. The XL1-Blue strain (Stratagene) was employed to propagate all other plasmids used in this work.

#### Cell culture

As described in,^11^ mouse embryonic fibroblasts (MEFs) used in this study had been isolated from 13.5-day-old embryos of WT and SPPL2a/b double-deficient mice. The head and internal organs of the embryos were removed, and the remaining tissue was dissected into small pieces. These were incubated in Trypsin-EDTA solution at 37°C for 15 minutes. Subsequently, the cells were dissociated by pipetting, and the resulting suspension was mixed with prewarmed DMEM supplemented with 10% FCS (fetal calf serum) and antibiotics. The cells were recovered by centrifugation, washed once with fresh medium, and plated for further culture. For MEF immortalization, an early passage of cells was transfected with an expression plasmid containing the SV40 large T antigen (pMSSVLT^84^). After approximately 10 passages, the cells became immortalized and were used for subsequent experiments. HEK293T CD9 KO and CD81 KO cells have been generated by Prof. Tom Gallagher (Loyola University, Chicago) as described in.^82^ HEK293T (DSMZ, Germany), HeLa (DSMZ), MEFs and Platinum-E retroviral packaging cells (Cell Biolabs Inc.) were cultivated in Dulbecco’s modified Eagle’s medium (DMEM, Gibco) supplemented with 10% fetal calf serum (FCS, Thermo Fisher Scientific) and 100 U/ml penicillin (Sigma-Aldrich) as well as 100 μg/ml streptomycin (Sigma-Aldrich). All cell lines were maintained at 37°C in a humidified 5% CO_2_–95% air atmosphere.

### Method details

#### RNA isolation and cDNA synthesis

To extract total RNA from mammalian cell cultures, the Nucleospin RNA Plus extraction kit (Macherey-Nagel) was used following the manufacturer's instructions. The concentration of the resulting RNA was determined spectro-photometrically. For preparation of 20 μl cDNA, 1 μg of isolated RNA was diluted in RNAse-free ddH_2_O to achieve a final volume of 11 μl and incubated at 65°C for 5 minutes with 1 μl of random hexamer primer. Subsequently, 4 μl of 5x Revert Aid reaction buffer, 1 μl of RiboLock RNase inhibitor, 2 μl of 10 mM dNTPs, and 1 μl of RevertAid reverse transcriptase (from the RevertAid First Strand cDNA Synthesis Kit, Thermo Fisher Scientific) were added. The reaction mix was incubated at 25°C for 5 minutes, followed by a 60-minute incubation step at 45°C for reverse transcription. Finally, the reverse transcription was terminated by incubating the samples at 70°C for 5 minutes. The cDNA was stored at -20°C until further use.

#### Cloning of plasmids

Generation of pcDNA3.1 Hygro(+) expression constructs encoding murine SPPL2a and SPPL2b fused to a C-terminal Myc-tag^52^ and murine Tumor necrosis factor (TNF) with an N-terminal HA and a C-terminal V5 epitope^83^ has been described before. A construct of the untagged p31 isoform of murine CD74 in the same vector was generated based on previously reported constructs.^85^ Therefore, the respective coding sequence was subcloned into pcDNA3.1 Hygro (+) via BamHI and XhoI restriction sites. As described above, the open reading frames (ORFs) of CD9, CD81, and CD82 were amplified from murine splenic cDNA. The coding sequence of a 3x-FLAG-epitope was fused to the 3’ end of the ORFs by PCR and the inserts were integrated into pMSCV puro vectors (Clontech) using BglII and XhoI restriction sites. Alternatively, the same ORFs were inserted into pcDNA3.1 Hygro (+) (Thermo Fisher Scientific), which had been prepared by digestion using BamHI and XhoI. The open reading frame of murine CADM1 (Cell adhesion molecule 1) was PCR-amplified from MEF cDNA and subcloned into pcDNA3.1 Hygro (+) via HindIII and NotI restriction sites creating an expression construct for a fusion protein with a C-terminally appended HA epitope. To generate an expression construct for a farnesylated form of GFP, the GFP ORF was amplified from peGFP-N1 (Clontech) and subcloned into pcDNA3.1 Hygro (+) via HindIII and XhoI sites. The ORF of the lysosomal membrane protein TMEM192 was recovered by PCR from IOH26789-pdEYFP-C1amp described in^86^ and integrated into peGFP-N1 by utilizing HindIII and BamHI sites. All oligonucleotides employed are listed above.

#### Transient transfection

For transient transfection of 80% confluent HEK293T, HeLa and Platinum-E cells, polyethylenimine (PEI MAX Transfection Grade Linear Polyethylene, Polysciences) was used in a 2.5:1 PEI/DNA ratio. The mixture was added to the cells to be transfected after incubation for 15 min at room temperature. Culture medium was replaced after 6 h. Cells were analysed 24 h after transfection.

#### Retroviral transduction

In order to stably transduce MEFs, Platinum-E cells were transfected with pMSCV constructs as described above. After 48 h, the virus-containing media were filtered through a 0.45 μm cell strainer (BD Biosciences) and transferred to the MEFs after being supplemented with 8 μg/ml polybrene (Sigma-Aldrich) for increasing the transduction efficiency. To select transduced cells, 10 μg/ml puromycin (Invivogen) was added 1 day after transduction. Transduced cells were maintained and used for experiments as polyclonal lines without any subcloning.

#### Generation of bone marrow-derived dendritic cells (BMDC)

The generation of BMDCs was based on the method described by Lutz et al.^15^^,^^87^ Bone marrow was flushed from the femur and tibia of wild type mice (C57BL/6 N Crl background) with BMDC medium (RPMI 1640 medium (Gibco) supplemented with 10% heat-inactivated FCS, 100 U/ml penicillin, 100 μg/ml streptomycin, and 50 μM β-mercaptoethanol). Bone marrow cells were dissociated by passing through a 23G cannula and a 100 μm cell strainer (Greiner Bio-One). The cells were seeded at a density of 5x10^6^ cells per 10 ml medium containing 20 ng/ml recombinant murine granulocyte-macrophage colony-stimulating factor (GM–CSF, Immunotools). After 3 days, 10 ml of fresh BMDC medium supplemented with 20 ng/ml of GM-CSF was added to the cells. On day 6, 10 ml of medium were removed and centrifuged at 210 g for 10 min. Sedimented cells were resuspended in 10 ml of BMDC medium with 10 ng/ml of GM-CSF and returned to the culture. At day 8 the BMDCs were harvested for experiments. Where indicated, BMDCs were treated with a combination of (Z-LL)_2_-ketone (40 μM, Peptanova) and inhibitor X (1 μM, Tocris) or E-64d (40 μM, Enzo). Stock solutions of these compounds were dissolved in DMSO.

#### Protein extraction and Western Blotting

For protein extraction from cultured cells, adherent cells were washed three times with phosphate-buffered saline (PBS), scraped off in PBS supplemented with 1x cOmplete protease inhibitor mix (Roche), and recovered by centrifugation. In case of BMDCs, the recovered suspension cells were combined with the adherent cells detached by scraping in PBS/cOmplete. Cells were centrifuged and washed in PBS and sedimented again. All cells pellets were resuspended in lysis buffer (50 mM Tris/HCl, pH 7.4, 150 mM NaCl, 1% (w/v) Triton X-100, 0.1% (w/v) SDS, and 4 mM EDTA) supplemented with 1x cOmplete protease inhibitor mix, 4 mM Pefabloc® SC Protease Inhibitor (Carl Roth) and 0.5 mg/ml Pepstatin A (Sigma-Aldrich) and incubated at 4°C for one hour followed by a centrifugation step at 18.000 g for 10 min. During the incubation, samples were sonicated at level 4 for 20 seconds at 4°C using a Branson Sonifier 450 (Emerson Industrial Automation). The concentration of protein lysates was measured using the Pierce BCA Protein Assay Kit (Thermo Fisher Scientific) according to the manufacturer’s recommendations. Prior to SDS-PAGE electrophoresis, samples were supplemented with 5x SDS sample buffer (500 mM dithiothreitol (DTT), 5% (w/v) SDS, 50% (v/v) glycerol, trace amounts bromophenol blue, and 625 mM Tris-HCl (pH 6.8)) or 4x Tricine loading buffer (150 mM Tris-HCl (pH 7.0), 40 mM DTT, 30% glycerol, 12% (w/v) SDS, and 0.05% (w/v) Coomassie Blue G-250) and were incubated either at 56°C for 10 min or 95°C for 5 min. Aliquots of protein lysates (20-40 μg) were subjected to SDS-PAGE electrophoretic separation using a standard Tris-glycine or Tris-tricine buffer depending on the protein to be detected. After electrophoresis, semidry transfer to a Whatman® Protran® nitrocellulose membrane with 0.2 μm pore size was performed as described.^50^ Transfer of Tris-glycine gels was conducted in 25 mM Tris, 192 mM glycine, 20% (v/v) methanol at 1 mA/cm^2^ for 2 h. For Tris-tricine gels, a transfer buffer with 300 mM Tris, 100 mM acetic acid, pH 8.6, was employed and gels were blotted for 16 h at 4°C with a constant current of 20 mA per gel. The membranes were blocked with 5% milk powder (Carl Roth) dissolved in Tris-buffered saline containing 0.1% (v/v) Tween-20 (TBST) for one hour. Incubation in primary antibody, which was diluted in the described blocking solution, was carried out overnight. Immunodetection was performed using the following antibodies: anti-mouse-CD9 (KMC8, Thermo Fisher Scientific, 1:1000), anti-human-CD9 (D3H4P, Cell Signaling Technology), anti-mouse-CD81 (D5O2Q, Cell Signaling Technology,1:1000), anti-human-CD81 (E2K9V, Cell Signaling Technology,1:1000), anti-Presenilin 1 (D39D1, Cell Signaling Technology,1:1000), anti-Cofilin (D3F9, Cell Signaling Technology,1:1000), anti-FLAG (M2, Sigma-Aldrich,1:1000), anti-HA (3F10, Roche,1:2000), anti-Na+/K+-ATPase (3010S, Cell Signaling Technology,1:1000), anti-Transferrin receptor (ab84036, Abcam,1:1000), anti-Transferrin receptor (clone H68.4, Cell Signaling Technology, 1:1000), anti-Actin (A2066, Sigma-Aldrich,1:4000), and anti-Connexin 43 (3512S, Cell Signaling Technology,1:1000). Polyclonal antisera against murine SPPL2a,^52^ human SPPL2a,^81^ murine SPPL2b^10^ and human SPPL2b^11^ have been described previously and were used in a dilution of 1:500. To allow detection of murine CD74, rabbits were immunised with a synthetic peptide (DDQRDLISNHEQLPILGNRPREPERC) corresponding to amino acids 2-27 within the cytoplasmic domain of the protein (Pineda Antikörper Service). Antibodies were affinity-purified against the immobilised immunogen. In addition, the established monoclonal antibody In-1 (BD Biosciences, 1:2000) directed against an N-terminal epitope of murine CD74 was employed. Secondary antibodies coupled to Horseradish peroxidase (HRP), were purchased from Dianova. HRP activity was visualised using an Amersham Imager 6000 upon the addition of ECL Western Blotting Detection Substrate (Bioquote). Images were processed with GNU Image Manipulation Program Software (GIMP, version 2.10.14) and analysed densitometrically using ImageJ software (version 1.52a).

#### Isolation of detergent-resistant membranes (DRMs)

For each preparation, cells from two 10-cm culture plates were harvested as described before and combined. Sedimented cells were resuspended in 500 μl Raft-buffer (20 mM HEPES NaOH pH 7.4, 150 mM NaCl, 1 mM EDTA) by five passages through a 25G cannula. After determining the protein concentration with the Pierce BCA Protein Assay Kit, equal protein amounts of each sample were adjusted to a total volume of 1 ml with Raft buffer and a final concentration of 1% of the respective detergent. For extraction with Triton X-100 and CHAPSO, samples were incubated for 30 min on ice. When Brij-98 was used, samples were kept at 37°C for 5 min followed by a 30 min incubation on ice. All employed buffer and detergent solutions were supplemented with 1x cOmplete protease inhibitor mix, 4 mM Pefabloc® SC Protease Inhibitor, and 0.5 mg/ml Pepstatin A. The lysates (1 ml) were adjusted to a sucrose concentration of 43% by mixing with 2 ml 65 % (w/v) sucrose in Raft buffer. To construct a discontinuous sucrose gradient, this sample was layered beneath 7 ml 35 % (w/v) and 3 ml 5 % (w/v) sucrose in Raft buffer, respectively. Separation was performed in an SW40Ti rotor (Beckman Coulter) at 263,627 x g_max_ for 16 h at 4°C. Fractions of 1 ml were collected from top to bottom. Aliquots of each fraction were supplemented with the required amount of 5x SDS sample buffer and analyzed by Western Blotting.

#### Isolation of total cellular membranes for membrane protein enrichment

Harvested and sedimented cells were briefly incubated in a hypotonic buffer (10 mM HEPES NaOH, 1 mM EDTA) to induce swelling and facilitate mechanical disruption by passage through a 27G cannula. Subsequently, homogenates were adjusted to final concentrations of 250 mM Sucrose, 10 mM HEPES-NaOH, pH 7.4, 1 mM EDTA and supplemented with protease inhibitors as described above. Nuclei were sedimented by centrifugation at 750 x g for 5 min. The resulting post-nuclear supernatant was subjected to ultracentrifugation at 100,000 x g for 1 h. The sedimented membranes were resuspended, adjusted to 0.1 M Na_2_CO_3_, pH 11.5, incubated on ice for 1 h according to,^88^ and centrifuged again as above. Membranes were washed once in 10 mM HEPES-NaOH, pH 7.4, and resuspended using sonication in the same buffer after final sedimentation. Protein concentration was determined using a BCA assay and samples were supplemented with the required amount of 5x SDS sample buffer to prepare them for electrophoretic separation.

#### Immobilization of antibodies on Sepharose beads

To couple the antibody directed against murine SPPL2b to Sepharose beads, 0.5 ml of antibody solution (50 % glycerol) was filtered through an Ultracel-30 regenerated cellulose membrane (Sigma-Aldrich) by centrifugation at 11.000 g to reduce the volume to 15 μl. After four washing steps with 500 μl coupling buffer (0.1 M NaHCO_3_, 0.5 M NaCl, pH 8.3) the antibody solution was recovered by centrifugation at 1000 g. The volume was adjusted to 50 μl using coupling buffer and the protein concentration was estimated spectrophotometrically with a Nanodrop2000c (Thermo Fisher Scientific). The antibody coupling step was performed according to the manufacturer’s recommendations using the Pierce™ Direct IP Kit (Thermo Fisher Scientific) and 2 mg of antibody.

#### Immunoprecipitation

Harvested and sedimented cells were lysed in IP buffer (50 mM Tris/HCl, pH 7.4, 150 mM NaCl, 4 mM EDTA and 1% (w/v) Brij-98 or 1% (w/v) Triton X-100 as indicated in the respective experiments) including protease inhibitors as described in the protein extraction section. In order to reduce non-specific binding to beads, a pre-adsorption step was performed with MEF lysates. For this purpose, CNBr Sepharose beads were blocked with TRIS in order to generate non protein-conjugated beads. MEF cell lysates were incubated with 50 μl of these sepharose beads for 1 h at 4°C. After sedimenting the beads, the supernatant/lysate was used for immuno-precipitation. For that, equal protein amounts of the lysates in a range of 0.75-1.5 mg were incubated with 25 μl of anti-FLAG M2 agarose gel (Sigma-Aldrich) or anti-SPPL2b Sepharose beads overnight at 4°C under constant rotation. Subsequently, after sedimenting beads by centrifugation at 6000 rpm for 1 min, beads were washed five times for 10 min with IP buffer. Immunocomplexes were eluted after the last washing step using 80 μl of 2x SDS sample buffer by incubation for 10 min at 56°C. Bound proteins were analysed by electrophoresis as described above.

#### Indirect immunofluorescence

For immunocytochemical stainings, HEK293 and HeLa cells were seeded on glass coverslips in 12 well plates and transiently transfected with 0.5 μg plasmid DNA on the subsequent day. One day after transfection, coverslips were washed three times with PBS and cells were fixed with 4% paraformaldehyde in PBS for 20 min at RT. The next steps included three washes with PBS/0,2 % saponine (Carl Roth), incubation for 5 min with 0.12% glycine/PBS supplemented with 0.2% saponine and for 1 h in blocking buffer (1% FCS in PBS/0,2 % saponine). Application of the primary antibody diluted in blocking buffer was performed at 4°C overnight. Overexpressed SPPL2 proteases and tetraspanins were detected based on their Myc or 3xFLAG epitope, respectively, using anti-Myc (71D10, Cell Signaling Technology, 1:200) and anti-FLAG (M2, Sigma-Aldrich, 1:200), respectively. In order to evaluate the subcellular localisation of SPPL2a, either endogenous Lysosome-associated membrane protein 2 (LAMP-2) was visualized with anti-LAMP-2^79^ or the lysosomal transmembrane protein 192 TMEM192^50^ was co-expressed as a GFP fusion protein. To label the plasma membrane in order to assess the localisation of SPPL2b, either the plasma membrane protein CADM1^89^ or a construct expressing GFP fused to a farnesyl anchor was co-expressed and visualised based on its C-terminally fused HA epitope using anti-HA (3F10, Roche) or GFP, respectively. For detection of CD74, the custom-made rabbit polyclonal antiserum described above was used. After five washes with PBS/0.2% saponine, the bound primary antibodies were visualised by employing Alexa 488-, 647-, and 594-conjugated secondary antibodies (Molecular Probes) diluted in blocking buffer. After 1 h of incubation, samples were washed 5 times with PBS/saponine followed by two washes with ddH_2_O. Afterwards, coverslips were mounted in Mowiol supplemented with 1,4-diazabicyclo[2.2.2]octane (DABCO) and 4′,6-diamidino-2-phenylindole (DAPI) on glass slides. Images were captured using a Leica Stellaris 8 confocal laser scanning microscope. Images were processed with ImageJ (version 1.52a) and GNU Image Manipulation Program software (GIMP, version 2.10.14). Analysis of co-localisation was performed using the JACop plugin in Image J.^90^ To ensure an unbiased analysis of the co-localisation, the Autothreshold plugin was used with “default” settings according to the original described method. To estimate the proportion of SPPL2a/b either co-localising with the respective analysed tetraspanin or a subcellular marker, Manders co-localisation coefficients (M1 and M2) were calculated for individual cells based on signals above the thresholds as detailed in the figure legends.

### Quantification and statistical analysis

Data are depicted as mean ± SD. The number of independent experiments (N) as well as the respective numbers of individual samples/replicates and thus individual data point (n) are indicated in the figure legends. Statistical significance was evaluated using unpaired two-tailed Student’s *t* test or one-way ANOVA followed by Tukey post hoc testing, as indicated. Significance levels of ∗p ,0.05, ∗∗p ,0.01, and ∗∗∗p ,0.001 were applied.

## Data Availability

• All data reported in this paper will be shared by the lead contact upon request. • This paper does not report original code. • Any additional information required to reanalyze the data presented in this paper will be made available by the lead contact upon request.
