# Supplementary material for: Dynamic association of the intramembrane proteases SPPL2a/b and their substrates with tetraspanin-enriched microdomains
Source: iScience. 2023 Sep 4;26(10):107819. doi: 10.1016/j.isci.2023.107819 (PMC10509304; doi:10.1016/j.isci.2023.107819)
Supplement: Document S1. Figures S1–S5 [file mmc1.pdf]

iScience, Volume *26*

Supplemental information

Dynamic association of the intramembrane proteases SPPL2a/b and their substrates with tetraspanin-enriched microdomains

Nadja Leinung, Torben Mentrup, Mehul Patel, Tom Gallagher, and Bernd Schröder

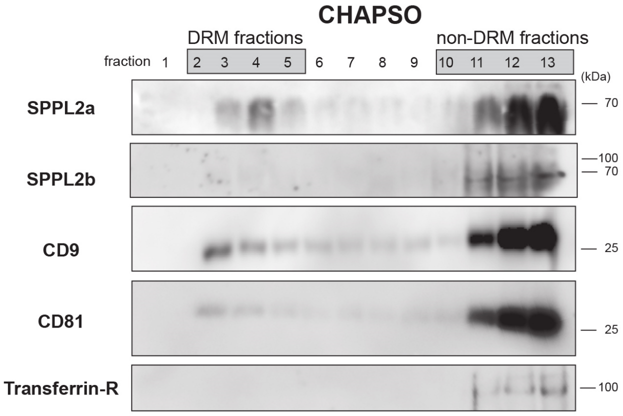

Figure S1, related to Figure 1. SPPL2a is partially associated with DRMs following CHAPSO extraction. MEFs from wild type mice were solubilised in 1% CHAPSO and subjected to a discontinuous sucrose density gradient at 263,627 x g_{max} for 16 h at 4°C. Thirteen fractions of 1 ml were collected starting from the top. Equal volumes of each fraction were analysed by Western blotting. The distribution of endogenous SPPL2a, SPPL2b, CD9, CD81, and the Transferrin receptor (Transferrin-R) in the gradient fractions was detected using specific antibodies.

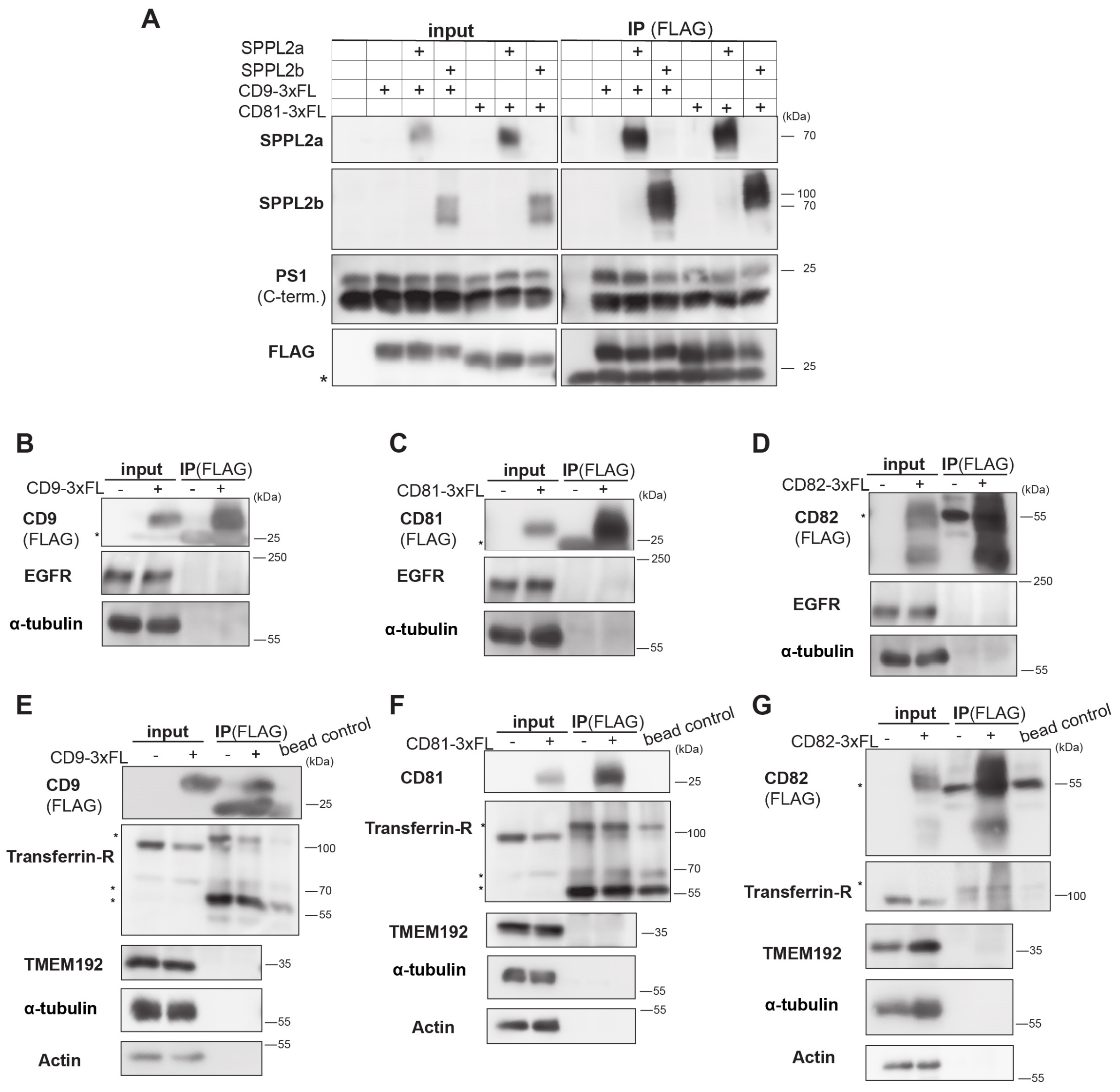

Figure S2, related to Figure 2. Presenilin-1 (PS1), but not unrelated control proteins, are co-immunoprecipitated with CD9 and CD81. **(A)** HEK cells were transiently transfected with expression constructs of SPPL2a, SPPL2b, CD9-3xFLAG or CD81-3xFLAG as indicated in the figure. Cells were lysed in 1% Brij-98, pulldown of FLAG-tagged CD9 or CD81 was performed using FLAG antibody-conjugated beads. Total lysates (input) and bead eluates (IP) were analysed by Western blotting using anti-FLAG, anti-Presenilin 1, anti-SPPL2a, and anti-SPPL2b. Asterisk, antibody band. (**B-D**) HEKs were transiently transfected with CD9-3xFLAG **(B)**, CD81-3xFLAG **(C)**, and CD82-3xFLAG **(D)**. After lysis with 1% Brij-98, lysates were subjected to immuno-precipitation with FLAG antibody-conjugated beads. Lysates (input) and bead eluates (IP) were subjected to Western blotting and specific antibodies directed against the epidermal growth factor receptor (EGFR) and α-tubulin were employed to examine the specificity of the FLAG-immuno-precipitation. Asterisk, antibody band**. (E-G)** MEF cells stably transduced with CD9-3xFLAG **(E)**, CD81-3xFLAG **(F)**, and CD82-3xFLAG **(G)** were lysed in 1% Brij-98. MEF lysates were subjected to pre-clearing steps employing non protein-conjugated sepharose beads prior to the immuno-precipitation by FLAG antibody-conjugated beads. Lysates (input) and bead eluates (IP) were subjected to Western blotting employing anti-FLAG, anti-CD81, anti-Transferrin receptor 1 (clone H68.4, Cell Signaling Technology), anti-Actin, anti-α-tubulin and anti-TMEM192 as control for the specificity of the FLAG-pulldown. Furthermore, a bead control was incorporated, where anti-FLAG beads without any lysate incubation were eluted and analysed in the same way as the IP samples. As the conjugated FLAG antibody as well as the antibody employed for detection of the murine transferrin receptor were both of mouse origin, specifically in this analysis we observed antibody bands, which were presumably the result of antibody fragments detached from the mouse antibody-coupled beads. Asterisk, antibody band.

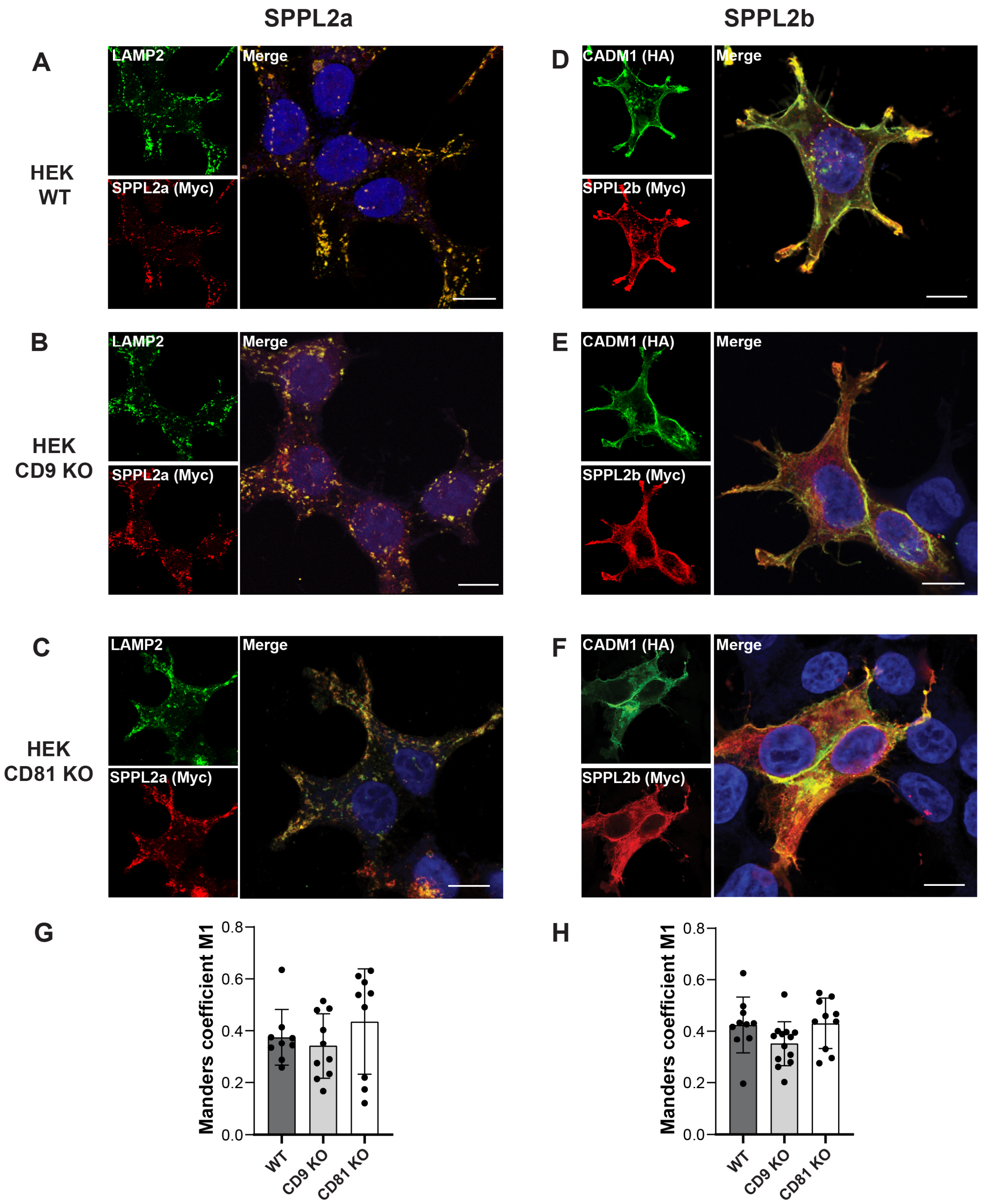

Figure S3, related to Figure 4. Subcellular targeting of SPPL2a/b is not affected in CD9- or CD81-deficient cells. Wild type (WT, **A**), CD9 KO (**B**) or CD81 KO (**C**) HEK cells were transiently transfected with expression constructs of SPPL2a-Myc or SPPL2b-Myc and HA-CADM1. After fixation, cells were analysed by indirect immunofluorescence. SPPL2a/b were detected based on their appended Myc epitope using anti-Myc. To analyse the subcellular localisation of SPPL2a, distribution of the endo/lysosomal marker LAMP-2 was visualised in parallel. To assess the enrichment of SPPL2b at the plasma membrane, cells were co-stained with anti-HA to label the co-expressed plasma membrane-localised protein CADM1 (Cell adhesion molecule 1). Scale bar, 10 µm. Co-localisation of SPPL2a/b with the respective sub-cellular markers was compared between WT, CD9, and CD81 KO cells using Fiji and the JACop plugin. Manders´coefficent M1 represents the overlap of the signals representing SPPL2a or SPPL2b staining with signals for the respective sub-cellular marker. Each data point represents analysis of the SPPL2a or SPPL2b-expressing cells, respectively, within one microscopic image. Mean ± S.D. N=2, n=9-13. A one-way ANOVA with Tukey’s post-hoc test was performed.

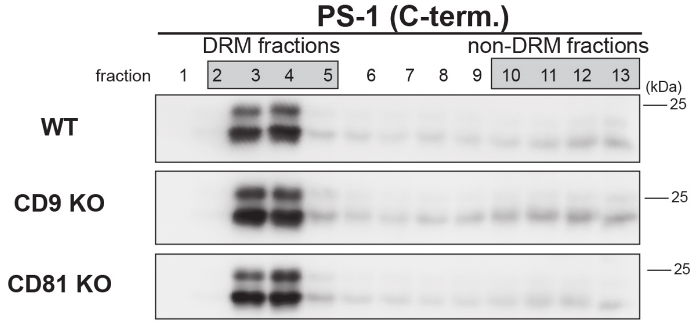

Figure S4, related to Figure 4. Association of Presenilin 1 with DRMs is not altered in CD9 and CD81 KO HEKs. Lysates of HEK WT, CD9 KO, and CD81 KO cells solubilized in 1% Brij-98 were separated on a discontinuous sucrose density gradient at 263,627 x g_{max} for 16 h at 4°C. Thirteen fractions of 1 ml were collected starting from the top. Equal volumes of each fraction were analysed by Western blotting. The distribution of Presenilin 1 was analyzed employing anti-Presenilin-1.

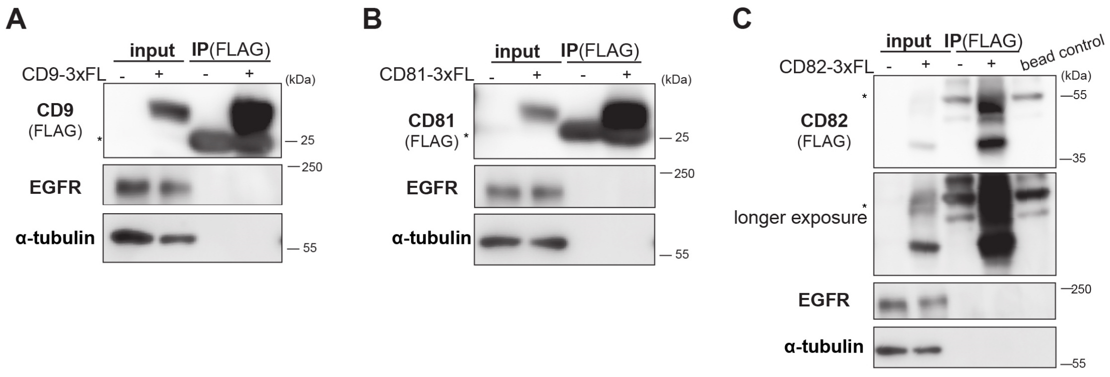

Figure S5, related to Figure 5. Control for specificity of FLAG-immunoprecipitation under Triton X-100 conditions. HEK cells were transfected with CD9-3xFLAG, CD81-3xFLAG, and CD82-3xFLAG constructs. Following cell lysis using 1% Triton X-100, the lysates were subjected to immunoprecipitation using FLAG antibody-conjugated beads. The lysates (input) and the eluates from the beads (IP) were subsequently analyzed through Western blotting. Specific antibodies targeting the epidermal growth factor receptor (EGFR) and α-tubulin were utilized to assess the specificity of the FLAG immunoprecipitation. The tetraspanins were detected with anti-FLAG. In (C) a bead control was incorporated, where “empty” beads without any incubation in the presence of lysates were eluted and analysed in the same way as the IP samples in order to identify bands in the FLAG detection just representing detached antibody. Asterisk, antibody band.
